# Mechanism of sphingolipid homeostasis revealed by structural analysis of *Arabidopsis* SPT-ORM1 complex

**DOI:** 10.1126/sciadv.adg0728

**Published:** 2023-03-29

**Authors:** Peng Liu, Tian Xie, Xinyue Wu, Gongshe Han, Sita D. Gupta, Zike Zhang, Jian Yue, Feitong Dong, Kenneth Gable, Somashekarappa Niranjanakumari, Wanyuan Li, Lin Wang, Wenchen Liu, Ruifeng Yao, Edgar B. Cahoon, Teresa M. Dunn, Xin Gong

**Affiliations:** ^1^ Department of Chemical Biology, Department of Biology, School of Life Sciences, Southern University of Science and Technology, Shenzhen, Guangdong 518055, China.; ^2^ Department of Biochemistry and Molecular Biology, Uniformed Services University of the Health Sciences, Bethesda, MD 20814, USA.; ^3^ State Key Laboratory of Chemo/Biosensing and Chemometrics, Hunan Provincial Key Laboratory of Plant Functional Genomics and Developmental Regulation, College of Biology, Hunan University, Changsha, Hunan 410082, China.; ^4^ Center for Plant Science Innovation and Department of Biochemistry, University of Nebraska-Lincoln, Lincoln, NE 68588, USA.

## Abstract

The serine palmitoyltransferase (SPT) complex catalyzes the first and rate-limiting step in sphingolipid biosynthesis in all eukaryotes. ORM/ORMDL proteins are negative regulators of SPT that respond to cellular sphingolipid levels. However, the molecular basis underlying ORM/ORMDL-dependent homeostatic regulation of SPT is not well understood. We determined the cryo–electron microscopy structure of *Arabidopsis* SPT-ORM1 complex, composed of LCB1, LCB2a, SPTssa, and ORM1, in an inhibited state. A ceramide molecule is sandwiched between ORM1 and LCB2a in the cytosolic membrane leaflet. Ceramide binding is critical for the ORM1-dependent SPT repression, and dihydroceramides and phytoceramides differentially affect this repression. A hybrid β sheet, formed by the amino termini of ORM1 and LCB2a and induced by ceramide binding, stabilizes the amino terminus of ORM1 in an inhibitory conformation. Our findings provide mechanistic insights into sphingolipid homeostatic regulation via the binding of ceramide to the SPT-ORM/ORMDL complex that may have implications for plant-specific processes such as the hypersensitive response for microbial pathogen resistance.

## Teaser

Structural analysis of *Arabidopsis* SPT-ORM1 complex reveals critical insights into sphingolipid homeostatic regulation.

## INTRODUCTION

**Fig. 1.**
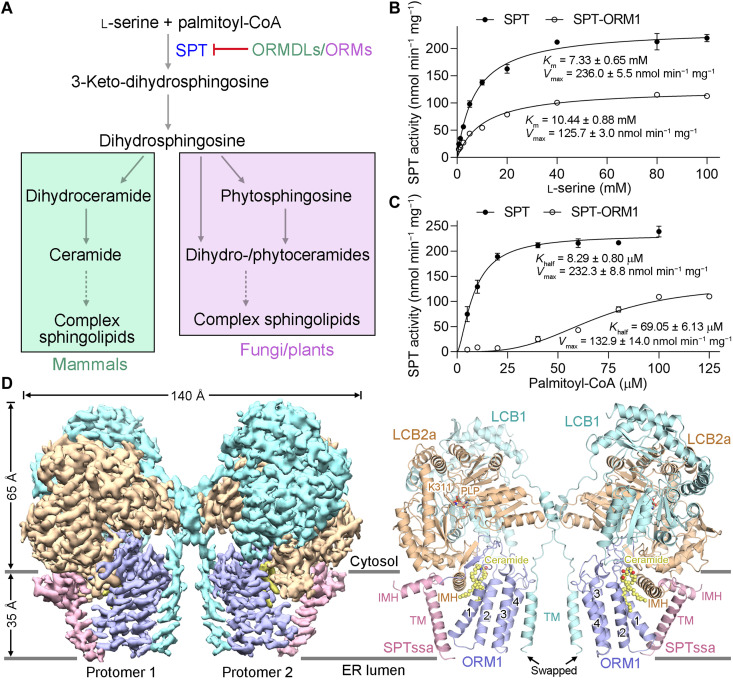
Biochemical characterization and structural determination of AtSPT-ORM1 complex. (**A**) A diagram of the de novo sphingolipid biosynthetic pathways in mammals, fungi, and plants. The serine palmitoyltransferase (SPT) activity is negatively regulated by ORMDLs/ORMs. (**B**) SPT activity versus l-serine concentration measured using SPT or SPT-ORM1 complex. The data points were fitted with a Michaelis-Menten equation. For all curves and dot plot graphs, each data point is the average of three independent experiments, and error bars indicate the SEM. (**C**) SPT activity versus palmitoyl–coenzyme A (CoA) concentration for SPT and SPT-ORM1 complex. The data points were fitted with an allosteric sigmoidal equation. (**D**) The cryo–electron microscopy (cryo-EM) map and overall structure of SPT-ORM1 complex. LCB1 and LCB2a are shown in light cyan and wheat, respectively; SPTssa and ORM1 are shown in pink and light blue, respectively; ceramide is shown in yellow. The cofactor pyridoxal 5′-phosphate (PLP) is shown in wheat sticks. TM, transmembrane helix; IMH, in-plane membrane helix; ER, endoplasmic reticulum. All structural figures were prepared using PyMOL (*59*) or UCSF Chimera (*60*).

Sphingolipids are ubiquitous lipid molecules as vital membrane components and bioactive signaling molecules in all eukaryotic cells (*1*–*3*). The sphingolipid levels need to be tightly controlled to preserve normal cellular functions (*4*, *5*). The sphingolipid metabolism pathways are generally conserved in animals, plants, and fungi (*3*, *6*, *7*). Sphingolipid biosynthesis begins in the endoplasmic reticulum (ER) with condensation of serine and palmitoyl–coenzyme A (CoA) by the serine palmitoyltransferase (SPT) complex (Fig. 1A) that forms long-chain base (LCB) components of sphingolipids. SPT has been regarded as the primary target for sphingolipid homeostatic regulation (*8*, *9*). This regulation is critical in plants to balance the production of glycosphingolipids, including glycosylinositolphosphoceramides and glucosylceramides, to support growth and the accumulation of programmed cell death (PCD)–inducing sphingolipid biosynthetic intermediates, including LCBs and ceramides, for fungal and bacterial pathogen resistance (*10*, *11*). Ceramides generated downstream by acylation of SPT-generated LCBs are precursors of all complex sphingolipids, occurring in plants as dihydroxy LCB-containing dihydroceramides and trihydroxy LCB-containing phytoceramides (Fig. 1A) (*12*).

SPT activity can be negatively regulated by a conserved family of small ER membrane–bound proteins, the ORM proteins in yeast and plants, and ORM-like (ORMDL) proteins in mammals (*13*–*18*). The ORM/ORMDL proteins form stable complexes with SPT (*14*, *15*). In yeast cells, several N-terminal serine residues of ORM proteins, which are absent from ORM/ORMDL proteins of multicellular eukaryotes (fig. S1A), can be phosphorylated by the Ypk kinases in response to reduced sphingolipid levels, thus releasing SPT from ORM-dependent inhibition (*14*, *19*, *20*). The elevated sphingolipid levels inhibit SPT activity in an ORM/ORMDL-dependent manner in both mammalian and yeast cells (*21*–*23*). In *Arabidopsis*, suppression of ORM-mediated regulation of SPT has been shown to result in marked accumulation of ceramides and large alterations in endomembranes and responses to bacterial pathogens and fungal mycotoxins (*16*, *17*, *24*). The recently reported structures of human SPT/SPT-ORMDL complexes in apo, substrate-bound, product-bound, and inhibitor-bound states reveal the overall assembly and substrate selectivity of this enzyme and give hints about the mechanism of SPT regulation by ORMDL (*25*–*27*). However, how ORM/ORMDL proteins adjust SPT activity in response to cellular sphingolipid levels remains largely enigmatic in all eukaryotes and especially in plants for the regulation of growth and responses to biotic and abiotic stresses.

## RESULTS

### Biochemical characterization of *Arabidopsis* SPT and SPT-ORM1 complexes

In *Arabidopsis*, the composition and function of SPT complex have been well established (*28*–*30*), and ORM1/2 has been demonstrated to interact with SPT and contribute to sphingolipid homeostasis (*16*, *17*, *24*). The protein sequences of *Arabidopsis* ORMs, lacking the N-terminal phosphorylation sites, are more similar to those of mammalian ORMDLs than to those of yeast ORMs (fig. S1A), suggesting that the mechanisms of ORM/ORMDL-dependent SPT regulation are more conserved in multicellular eukaryotes (plants and mammals) than in yeast. Likewise, the protein sequences of the small subunits of the SPT complex are more conserved in multicellular eukaryotes compared to yeast (fig. S1B).

The recombinant protein expression and purification of *Arabidopsis* SPT (AtSPT; composed of LCB1, LCB2a, and SPTssa) and SPT-ORM1 (AtSPT-ORM1; composed of LCB1, LCB2a, SPTssa, and ORM1) complexes in human embryonic kidney (HEK) 293F cells are described in detail in Materials and Methods. The purified SPT and SPT-ORM1 complexes are catalytically active and can be repressed by a potent SPT-specific inhibitor myriocin (Fig. 1, B and C; and fig. S2, A and B), suggesting that the functional SPT/SPT-ORM1 complexes can be obtained from this mammalian expression system. The ORM1-dependent SPT inhibition can be demonstrated with the purified proteins, as the specific activity of the SPT-ORM1 complex at saturating conditions is only around 50% of that of the SPT complex (Fig. 1, B and C). The *K*_half_ value of SPT-ORM1 complex toward palmitoyl-CoA is nearly eight times as high as that of the SPT complex (Fig. 1C), implying that the association of SPT with ORM1 could markedly decrease the affinity of SPT toward palmitoyl-CoA.

### Structural determination of the AtSPT-ORM1 complex

To explore the structural basis of SPT inhibition by ORM1, we sought to determine the structures of SPT and SPT-ORM1 complexes by single-particle cryo–electron microscopy (cryo-EM). However, the SPT complex sample appears highly heterogeneous in the cryo-EM image (fig. S2C), impeding the high-resolution reconstruction of the complex. By contrast, the SPT-ORM1 complex shows homogeneous structural characteristics in the representative two-dimensional (2D) averages (fig. S2D), suggesting an important role of ORM1 in stabilizing the SPT assembly. A dimeric organization of the SPT-ORM1 complex was observed in the 2D averages (fig. S2D), resembling the reported structures of the human SPT-ORMDL3 complex (*25*, *26*).

The cryo-EM reconstructions of the dimeric and monomeric SPT-ORM1 complex were both obtained at an overall resolution of 3.2 Å (fig. S3), allowing accurate model building for most of the protein complex (fig. S4). Each protomer of the SPT-ORM1 dimer consists of LCB1, LCB2a, SPTssa, and ORM1 at a 1:1:1:1 ratio, and the transmembrane (TM) helix of LCB1 is swapped (Fig. 1D). The final atomic model contains 1120 amino acids for the monomeric complex, including 445 from LCB1 (residues 36 to 480), 471 from LCB2a (residues 1 to 36 and 41 to 475), 48 from SPTssa (residues 1 to 48), and 156 from ORM1 (residues 1 to 156) (figs. S1 and S5 and table S1). In each protomer, a pyridoxal 5′-phosphate (PLP) cofactor was covalently linked to the ε-amino group of Lys^311^ in LCB2b as an internal aldimine (Fig. 1D). In addition, a robust lipid-like density that might belong to ceramide could be unambiguously resolved between ORM1 and LCB2a in the cytosolic membrane leaflet (Fig. 1D), which will be further discussed below.

The overall structure of the AtSPT-ORM1 complex is quite similar to that of the human SPT-ORMDL3 complex in both dimeric and monomeric forms, except for some local movements in the TM regions (fig. S6, A and B). Consistent with the high levels of conservation between *Arabidopsis* and human SPT/ORM sequences (figs. S1 and S5), the structures of the four single subunits are highly conserved, except for some differences at the N- and C-terminal regions (fig. S6C).

### A functional ceramide binding site

**Fig. 2.**
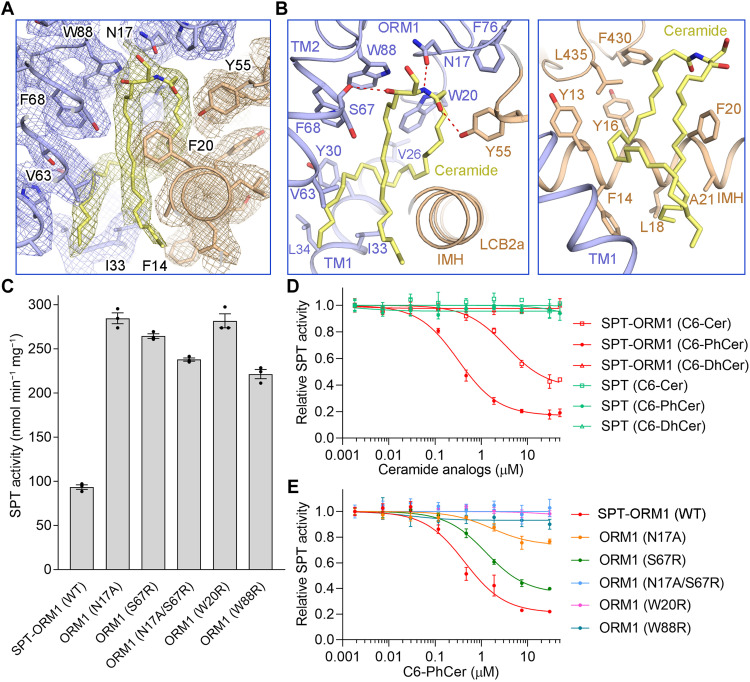
The ceramide binding site in AtSPT-ORM1 complex. (**A**) A close-up view of the electron density map for the ceramide binding site contoured at 5σ. (**B**) Detailed views of the ceramide binding site between ORM1 and LCB2a. The ceramide-binding residues are shown as sticks. All potential polar interactions are displayed by red dashed lines. (**C**) SPT activity of the SPT-ORM1 variants bearing mutations in the ceramide-binding site. Enzyme activity was determined with saturating concentrations of substrates. WT, wild type. (**D**) The inhibition curves of SPT and SPT-ORM1 complexes by C6-ceramide (C6-Cer), C6-phytoceramide (C6-PhCer), and C6-dihydroceramide (C6-DhCer) in the normalized SPT activity assay. All the estimated half-maximal inhibitory concentration (IC_50_) values were summarized in table S2. (**E**) Inhibition curves of the SPT-ORM1 ceramide-binding variants by C6-PhCer.

During structural determination, a clear lipid-like density was observed in the cytosolic membrane leaflet sandwiched between ORM1 and LCB2a (Fig. 2A). This lipid-like density can be well fitted with a ceramide molecule and was tentatively assigned as C24-ceramide (d18:1/24:0) in the structure (Fig. 2A and fig. S4B). The ceramide is presumably derived from endogenous ceramide copurifying with the SPT-ORM1 complex. To corroborate this notion, lipids in the purified SPT-ORM1 complex were extracted and subjected to liquid chromatography–tandem mass spectrometry (LC-MS/MS) and thin-layer chromatography (TLC) analysis. The results confirmed the presence of ceramide in the SPT-ORM1 complex (fig. S7, A and B).

The small polar head of C24-ceramide forms potential hydrogen bond interactions with the side-chain groups of Asn^17^ in ORM1 and Tyr^55^ in LCB2a and the main chain group of Ser^67^ in ORM1 (Fig. 2B). The aliphatic sphingosine and acyl chains of ceramide are coordinated by numerous hydrophobic residues from both ORM1 and LCB2a (Fig. 2B). To probe the function of residues that participate in ceramide coordination, we performed structure-guided mutational analyses. All the SPT-ORM1 variants were individually purified to ensure their homogeneity (fig. S8A) and examined by enzymatic assays with saturating concentrations of substrates. All the variants, including ORM1-N17A, designed to weaken the coordination of ceramide polar head, and ORM1-S67R, ORM1-W20R, and ORM1-W88R, designed to repel ceramide aliphatic chains, exhibited greatly increased enzymatic activity compared to the wild-type (WT) SPT-ORM1 complex (Fig. 2C), supporting the importance of ceramide binding residues for ORM1-dependent SPT repression.

It has been demonstrated that SPT activity can be inhibited by exogenously added short-chain ceramide analogs in an ORM/ORMDL-dependent manner for both mammalian and yeast proteins in experiments performed in cells or crude membrane extracts (*21*–*23*). Consistent with this, the enzymatic activity of the purified SPT-ORM1 complex could be further inhibited to around 40% by exogenously added C6-ceramide with a half-maximal inhibitory concentration (IC_50_) of 3.5 μM (Fig. 2D, fig. S7C, and table S2). C6-phytoceramide, a short-chain analog of the predominant trihydroxy LCB-containing *Arabidopsis* ceramide, has a stronger inhibitory effect (maximally inhibited to around 20% with an IC_50_ of 0.32 μM) than does C6-ceramide, while C6-dihydroceramide has no apparent effect on the SPT-ORM1 complex (Fig. 2D). The results suggest that there is specificity for the ceramide species that can be sensed by SPT-ORM1 complex. In the absence of ORM1, the SPT complex does not respond to C6-ceramide, C6-phytoceramide, or C6-dihydroceramide (Fig. 2D), confirming that the binding of ceramide analogs to the SPT complex is ORM1 dependent.

To explore whether the exogenously added C6-phytoceramide binds to the ceramide binding site revealed in our structure, we examined the responsiveness of the ceramide binding variants to C6-phytoceramide. The ORM1-N17A or ORM1-S67R mutation lowered the inhibitory potency of C6-phytoceramide (Fig. 2E), suggesting partially disrupted C6-phytoceramide binding. The ORM1-N17A/S67R double mutation nearly eliminated the inhibitory effect of C6-phytoceramide (Fig. 2E), indicating largely disrupted C6-phytoceramide binding. The ORM1-W20R or ORM1-W88R mutation also virtually abolished the inhibitory effect of C6-phytoceramide (Fig. 2E). Together, these results indicate that ceramide binding is important for ORM1-dependent SPT repression and that exogenously added C6-phytoceramide can mediate the ORM1-dependent SPT repression via the structurally revealed ceramide binding site.

Although the ceramide binding variant SPT-ORM1 (ORM1-N17A) exhibited a very close *V*_max_ value compared to the WT SPT complex, the *K*_half_ value of the variant toward palmitoyl-CoA was nearly seven times as high as that of the WT SPT complex (fig. S8C), suggesting that, in the absence of ceramide binding, ORM1 can still repress SPT by competitive inhibition that decreases the affinity of SPT toward palmitoyl-CoA. The WT SPT-ORM1 complex represents a ceramide-bound state and has a decreased *V*_max_ and an increased *K*_half_ for palmitoyl-CoA compared to the ceramide-binding variant SPT-ORM1 (ORM1-N17A), indicating that the ceramide binding can repress SPT-ORM1 by mixed inhibition.

To further examine the impact of ceramide binding residues on ORM1-mediated regulation of SPT activity in a cellular environment, the *Arabidopsis* ORM1 mutants were coexpressed with AtSPT (LCB1, LCB2a, and Flag-SPTssa) in HEK293 *SPTSSA* knockout (KO) cells and incorporation of d2-labeled serine into de novo–synthesized LCBs was measured (fig. S9). Expression of WT ORM1 substantially reduced intracellular SPT activity, implying that the regulation of AtSPT by AtORM1 can be recapitulated in HEK293 cells. We showed that ORM1-W20R and ORM1-W88R markedly impaired the regulation of intracellular SPT activity, while ORM1-N17A/S67R only modestly affected regulation (fig. S9). Although the in vitro data showed that the purified AtSPT-ORM1 complex containing the ORM1-N17A/S67R mutation was barely inhibited by C6-PhCer, the complex was still inhibited in HEK cells. This likely reflects that the native long-chain (C14 to C26) ceramides contribute to stronger hydrophobic interactions than the C6-PhCer as well as the fact that ceramide levels are extremely elevated because of overexpression of AtSPT. These results indicate that the ceramide-binding residues in ORM1, especially Trp^20^ and Trp^88^, are important for ORM1-mediated SPT regulation in cells. Note that HEK293 cells were used as an experimentally amenable proxy for plant cells to investigate the AtSPT regulation by AtORM1 in a cellular environment. However, in planta regulation of AtSPT by key AtORM1 mutants, informed by these structural studies, will be conducted in the future.

### A hybrid β sheet between the N termini of ORM1 and LCB2a

**Fig. 3.**
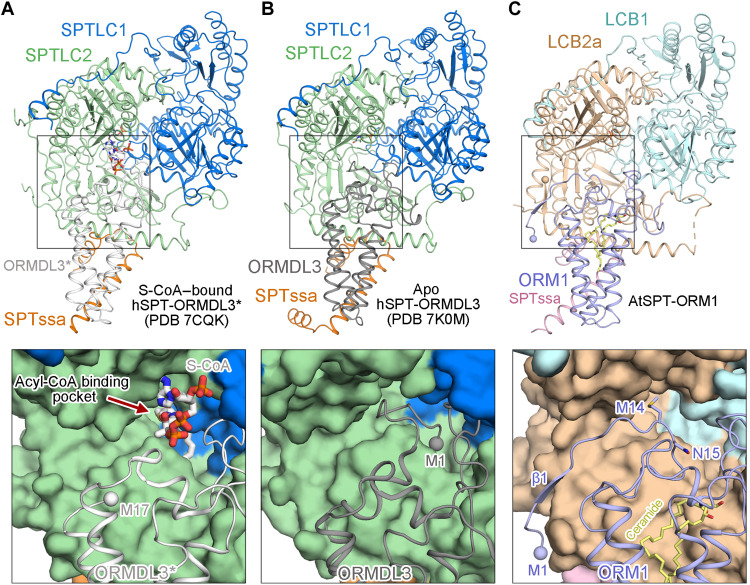
Distinct conformations of the N termini of AtORM1 and hORMDL3. (**A**) The N terminus of hORMDL3 appears highly flexible in the previously reported acyl-CoA–bound hSPT-ORMDL3* complex structure [Protein Data Bank (PDB) 7CQK]. SPTLC1 and SPTLC2 are colored marine and light green, respectively; SPTssa and ORMDL3* are colored orange and light gray, respectively. *S*-(2-oxoheptadecyl)–CoA (S-CoA) is shown in gray sticks. (**B**) The N terminus of hORMDL3 inserts into the acyl-CoA binding pocket in the apo hSPT-ORMDL3 structure (PDB 7K0M). ORMDL3 is colored dark gray; all the other subunits are shown in the same color scheme as (A). (**C**) The N terminus of AtORM1 forms a β sheet with LCB2a in the *Arabidopsis* SPT-ORM1 (AtSPT-ORM1) complex structure. The potential acyl-CoA binding pocket in the AtSPT-ORM1 structure is partially shielded by Met^14^ and Asn^15^ from ORM1.

**Fig. 4.**
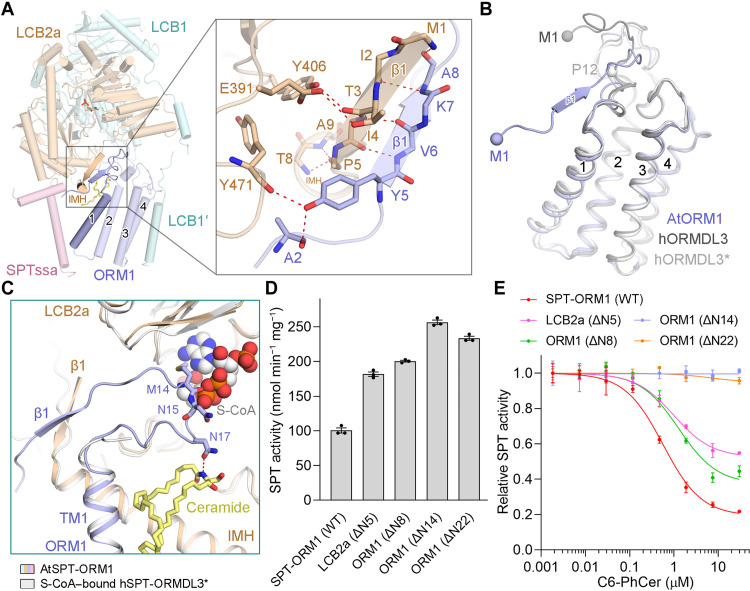
The hybrid β sheet between ORM1 and LCB2a stabilizes the ORM1 N terminus in an inhibitory conformation. (**A**) A close-up view of the hybrid β sheet between the N termini of ORM1 and LCB2a. (**B**) Comparison of AtORM1 and hORMDL3. The hORMDL3 subunit in the previously resolved SPT-ORMDL3 complex structures (PDB 7K0M and 6M4O) is named hORMDL3 and hORMDL3^*^, respectively. (**C**) The CoA head of the potential acyl-CoA substrate clashes with Met^14^ and Asn^15^ of ORM1 in the AtSPT-ORM1 structure. The potential acyl-CoA binding site was implied by the superimposition of the AtSPT-ORM1 structure and the S-CoA–bound hSPT-ORMDL3^*^ structure (PDB 7CQK). (**D**) SPT activity of the SPT-ORM1 variants with N-terminal deletion of LCB2a or ORM1. (**E**) Inhibition curves of the SPT-ORM1 variants with N-terminal deletion of LCB2a or ORM1 by C6-PhCer.

The N terminus of human ORMDL3 (hORMDL3) either appears highly flexible in our previously reported hSPT-ORMDL3^*^ complex structure (Fig. 3A) (*25*) or inserts into the acyl-CoA binding pocket in the hSPT-ORMDL3 structure reported byWang *et al.* (*26*) (Fig. 3B). Unlike either of these, the N terminus of AtORM1 (residues 5 to 8) forms an antiparallel hybrid β sheet with the N terminus of LCB2a (residues 1 to 5) in the AtSPT-ORM1 complex structure (Figs. 3C and 4A). The sequences of ORM/ORMDL proteins from different species are most divergent at their N termini (fig. S1A). Except for the dissimilarities at the N termini, the structures of AtORM1 and hORMDL3/hORMDL3^*^ can be superimposed with a root mean square deviation of 0.956/1.026 Å over 120/119 aligned Cα atoms (Fig. 4B).

We attempted to capture a substrate-bound structure of the AtSPT-ORM1 complex by incubating the protein with excess l-serine and *S*-(2-oxoheptadecyl)–CoA (S-CoA), a nonreactive palmitoyl-CoA analog, as we did for the hSPT-ORMDL3 complex (*25*). Despite numerous trials, we failed to observe any S-CoA density in the AtSPT-ORM1 complex structure. However, the substrate-binding residues in hSPT-ORMDL3 complex are nearly invariable in AtSPT sequences (fig. S10, A and B), implying that the substrate-binding sites are highly conserved across phyla. Superimposition of the substrate-bound hSPT-ORMDL3 structure [Protein Data Bank (PDB) 7CQK] and the AtSPT-ORM1 structure revealed the potential acyl-CoA binding site in the AtSPT-ORM1 complex (Fig. 4C).The CoA head of the acyl-CoA substrate clashes with Met^14^ and Asn^15^ of ORM1 in the AtSPT-ORM1 complex structure (Fig. 4C), suggesting that the current AtSPT-ORM1 structure might represent an inhibitory conformation that cannot bind the acyl-CoA substrate. This inhibitory conformation is consistent with the decreased activity of the AtSPT-ORM1 complex compared to that of the AtSPT complex (Fig. 1, B and C).

To investigate the function of the hybrid β sheet formed between ORM1 and LCB2a, we generated several AtSPT-ORM1 variants containing N-terminal deletions of LCB2a or ORM1 (fig. S8B). The ORM1-dependent SPT repression is largely relieved by LCB2a-ΔN5 (deletion of residues 2 to 5) or ORM1-ΔN8 (deletion of residues 2 to 8) (Fig. 4D). The LCB2a-ΔN5 and ORM1-ΔN8 variants were inhibited by C6-phytoceramide to a lesser degree than the WT AtSPT-ORM1 complex (Fig. 4E). These results suggest that the hybrid β sheet between ORM1 and LCB2a strengthens but is not absolutely required for the ORM1-dependent SPT inhibition. Nevertheless, when we further truncated the N-terminal sequence of ORM1, we found that the ORM1-ΔN14 (deletion of residues 2 to 14) or ORM1-ΔN22 (deletion of residues 2 to 22) mutation not only abolished the ORM1-dependent SPT inhibition (Fig. 4D) but also basically eliminated the inhibitory effect of C6-phytoceramide (Fig. 4E), indicating that the whole N-terminal region of ORM1 is indispensable for ORM1-dependent SPT inhibition. Consistent with this, the *K*_half_ value of the AtSPT-ORM1 (ORM1-ΔN14) variant toward palmitoyl-CoA was much lower than that of the WT AtSPT-ORM1 complex (fig. S8D), indicating that the N-terminal region of ORM1 is mainly responsible for the decreased affinity toward palmitoyl-CoA for the WT AtSPT-ORM1 complex. Together, these results imply that the hybrid β sheet between ORM1 and LCB2a is important for stabilizing the ORM1 N terminus in an inhibitory conformation.

We also investigated the impact of the N terminus of AtORM1 on ORM1-mediated regulation of SPT activity in the HEK293 *SPTSSA* KO cells (fig. S11). Expression of ORM1-ΔN14 repressed SPT activity, although to a much lesser extent than WT ORM1 (fig. S11A). Although the ORM1-ΔN14 mutant did not respond to the short-chain C6-PhCer in vitro (Fig. 4E), we suggest that, with an excessive amount of long-chain ceramides in the cells, the ORM1-ΔN14 mutant complex is still able to bind to long-chain ceramides in cells. The binding of long-chain ceramides might be sufficient to stabilize the Met^14^ and Asn^15^ of ORM1-ΔN14 in an inhibitory conformation and mediate the SPT inhibition in cells (fig. S11A). In addition, expression of ORM1-ΔN22 had no notable effect on intracellular SPT activity (fig. S11A), which supports the importance of the whole N-terminal 22 residues of AtORM1 for the regulation of intracellular SPT activity.

### Induced formation of the hybrid β sheet by ceramide binding

**Fig. 5.**
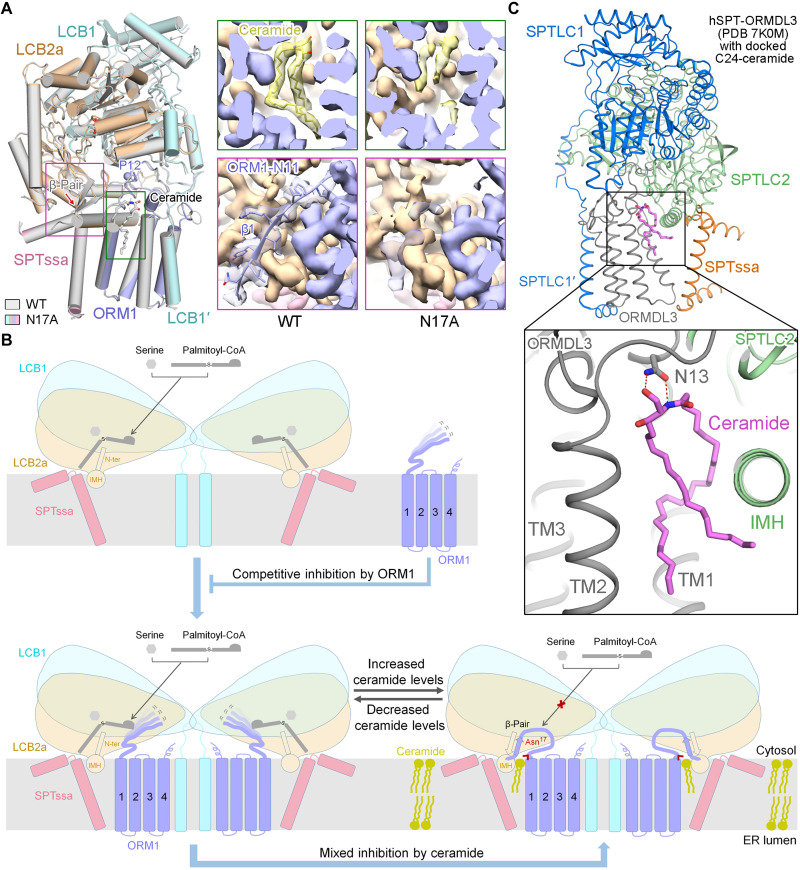
The formation of a hybrid β sheet between the N termini of ORM1 and LCB2a is induced by ceramide binding and a working model for SPT regulation by ORM1 and ceramide. (**A**) Superposition of the WT SPT-ORM1 structure (gray) and the ORM1-N17A mutant structure (colored based on the subunits). Pro^12^, the resolved N terminus of ORM1-N17A, is shown in a light blue sphere. Insets: Comparison of the EM maps for ceramide (top) and ORM1 N terminus (bottom) in the WT SPT-ORM1 and the ORM1-N17A mutant. The ceramide-like density largely diminished in the EM map of the ORM1-N17A mutant. The ORM1 N terminus (residues 1 to 11) is almost invisible in the ORM1-N17A mutant. (**B**) A working model for SPT regulation by ORM1 and ceramide. ORM1 competitively inhibits SPT using its N terminus to decrease the affinity of SPT toward palmitoyl-CoA. The SPT-ORM1 complex is a ceramide sensor in the ER that controls cellular sphingolipid homeostasis. When ceramide levels are insufficient, the ORM1 N terminus is flexible and allows the substrate entry for SPT catalysis, which would eventually lead to the accumulation of ceramide. With sufficient ceramide levels, the binding of ceramide with SPT-ORM1 induces the formation of a hybrid β sheet between the N termini of ORM1 and LCB2a, which stabilizes the ORM1 N terminus in an inhibitory conformation that blocks substrate entry. The Asn^17^ of ORM1, which contributes to the coordination of the polar head of ceramide and is strictly conserved across species, is highlighted. (**C**) The docked model of C24-ceramide bound with hSPT-ORMDL3 (PDB 7K0M) by PatchDock. The docked ceramide is shown in magenta sticks. The conserved Asn^13^ in hORMDL3 makes potential hydrogen bond interactions with the polar head of ceramide in the docked model.

To obtain the structure of the AtSPT-ORM1 complex in the absence of ceramide, we determined the structure of the ORM1-N17A variant at an overall resolution of 3.4 Å for the monomeric complex (fig. S12). The overall structure of the ORM1-N17A variant is essentially identical to that of the WT SPT-ORM1 complex, except for two apparent local structural differences (Fig. 5A). As anticipated, the density corresponding to ceramide greatly diminished in the ORM1-N17A variant (Fig. 5A), confirming the largely disrupted ceramide binding by the mutation. The other evident structural difference is that the density corresponding to the ORM1 N terminus (residues 1 to 11, referred to as ORM1-N11) mostly disappeared without forming the hybrid β sheet in the ORM1-N17A variant (Fig. 5A), probably because of the flexible nature of the ORM1 N terminus in the absence of ceramide. The flexible ORM1 N terminus might not be able to suppress the acyl-CoA substrate entry efficiently, thus largely releasing the ORM1-dependent SPT inhibition at saturating substrate concentrations. Our results suggest that ceramide binding could induce the formation of a hybrid β sheet between the N termini of ORM1 and LCB2a, thus stabilizing the ORM1 N terminus in an inhibitory conformation that blocks substrate binding.

We also determined the structure of SPT-ORM1 (LCB2a-ΔN5) variant at an overall resolution of 2.8 Å for the monomeric complex (fig. S13, A to C). In the LCB2a-ΔN5 mutant structure, a similar ceramide-like density could be resolved between ORM1 and LCB2a, but the N terminus of ORM1 (residues 1 to 10) appeared to be flexible (fig. S13, D and E). The flexible ORM1 N terminus is consistent with the greatly diminished ORM1-dependent SPT suppression for the LCB2a-ΔN5 variant (Fig. 4D). Together, these results suggest that both ceramide binding and the formation of a hybrid β sheet are critical for stabilizing the inhibitory conformation of the ORM1 N terminus.

## DISCUSSION

Our results provide critical insights into the regulation of AtSPT by ORM1 and ceramide (Fig. 5B). Our study suggests that ORM1 can competitively inhibit SPT using its N terminus to decrease the affinity of SPT toward palmitoyl-CoA substrate. Our study indicates that the SPT-ORM1 complex is a ceramide sensor in the ER that controls cellular sphingolipid homeostasis. When the ceramide levels are insufficient, the N terminus of ORM1 is highly mobile and cannot restrain substrate entry efficiently, thereby releasing the ceramide-mediated ORM1-dependent SPT suppression to enhance sphingolipid biosynthesis (Fig. 5B, bottom left). As the sphingolipid biosynthesis increases, ceramides accumulate above levels required for cellular functions. Then, ceramide would bind to the SPT-ORM1 complex and induce the formation of a hybrid β sheet between the N termini of ORM1 and LCB2a (Fig. 5B, bottom right). The hybrid β sheet stabilizes the N terminus of ORM1 in an inhibitory conformation that efficiently blocks substrate entry, thus down-regulating sphingolipid production (Fig. 5B, bottom right). In *Arabidopsis*, ceramide-induced SPT regulation balances the requirement of sphingolipids for growth and basal cellular functions while mitigating the accumulation of PCD-inducing biosynthetic intermediates (LCBs and ceramides), until needed for processes such as pathogen resistance.

A similar ceramide-binding cavity could be observed between ORMDL3 and SPTLC2 in the previously resolved hSPT-ORMDL3 structures (fig. S14), and one molecule of C24-ceramide fits nicely into this cavity by PatchDock (*31*) analysis (Fig. 5C). In the docking model, the Asn^13^ of hORMDL3, equivalent to the Asn^17^ of AtORM1, also makes potential hydrogen bond interactions with the polar head of ceramide (Fig. 5C). This asparagine residue is strictly conserved in the ORM/ORMDL sequences of all species (fig. S1A). Meanwhile, the mammalian and yeast SPT-ORM/ORMDL complexes can be inhibited by ceramide directly in isolated membranes (*21*–*23*). These facts together suggest that the ceramide-sensing mechanism proposed for the AtSPT-ORM1 complex (Fig. 5B) is also present in SPT-ORM/ORMDL complexes from other species.

Our study demonstrates that upon ceramide binding, the N terminus of AtORM1 forms a hybrid β sheet with the N terminus of LCB2a, thus stabilizing the Met^14^/Asn^15^ of AtORM1 to limit acyl-CoA binding (Fig. 4C). It has been suspected that the N terminus of hORMDL3 could project into the acyl-CoA binding pocket to reduce the SPT activity (Fig. 3B) (*26*). The N-terminal sequences of yeast ORMs are much longer than those of *Arabidopsis* and human ORMs/ORMDLs and contain the phosphorylation sites that are critical for the homeostatic regulation of yeast SPT activity (fig. S1A). Further studies are required to ascertain the precise mechanism of SPT regulation by ORM/ORMDL proteins of different species.

Our finding that C6-phytoceramides (i.e., trihydroxy LCB-containing ceramides), but not C6-dihydroceramides (i.e., dihydroxy LCB-containing ceramides), are effective at ORM1-mediated suppression of SPT activity is consistent with previous findings that suggested differential regulation of SPT by phyto- versus dihydroceramides (fig. S15). Consistent with strongly elevated SPT activity, *Arabidopsis* engineered for *LOH2* overexpression or KO of *sbh* genes encoding the LCB C4-hydroxylase primarily produce C16-dihydroceramides and display hyperaccumulation of sphingolipids by amounts ≤3-fold than those found in WT plants (fig. S15) (*32*, *33*). By contrast, overexpression of *LOH1* or *LOH3*, class II ceramide synthases that preferentially generate C24-phytoceramides, does not result in hyperaccumulation of sphingolipids (*33*). Together, these results are consistent with the observation that C6-phytoceramide, but not C6-dihydroceramide, mediates the effective ORM-dependent inhibition of AtSPT.

Note that the strongly elevated accumulation of C16-dihydroceramides in the *sbh* mutants and *LOH2* overexpressors is accompanied by the accumulation of salicylic acid and the triggering of PCD (*32*, *33*), hallmarks of the hypersensitive response for microbial pathogen resistance (*34*–*36*). Collectively, our findings and those from previous studies (*32*, *33*, *37*) point to the possibility that the relatively low SPT inhibitory effect of dihydroceramides from class I ceramide synthase allows plants to accumulate ceramides needed to mount and sustain the hypersensitive response for microbial pathogen resistance (fig. S15). Note that partial SPT inhibition was observed in our studies with C6-ceramides, containing sphingosine, a C18 Δ4 unsaturated dihydroxy LCB, which is found in only small amounts in plants (*38*). This suggests that hydroxylation or unsaturation at the C-4 position of LCBs contributes to the ceramide-induced ORM1-dependent suppression of SPT activity. Studies of SPT activity with additional ceramide species are needed to clarify the structural features of ceramides, including fatty acid chain length (e.g., C16 versus very long-chain fatty acids), which contribute to sphingolipid homeostatic regulation and microbial pathogen resistance. It will also be interesting to investigate whether differential ORM-mediated repression of SPT by distinct ceramides extends to other species.

Cells demand a series of regulatory mechanisms to maintain appropriate levels of various lipid species, such as sterols, glycerophospholipids, and sphingolipids. The pioneering work from the Brown and Goldstein laboratory (*39*, *40*) has revealed critical insights into the mechanism of sterol sensing by membrane-embedded protein sensors, such as Scap and Insigs. The above sterol sensors are regulatory proteins that can monitor the sterol concentrations in the ER and then mediate sterol-regulated transcriptional activation of sterol synthesis and uptake genes (*41*, *42*) or sterol-triggered degradation of sterol synthesis enzyme (*43*). Distinct from the sterol sensors mentioned above, the ceramide sensor presented here is an upstream sphingolipid biosynthetic enzyme complex. It will be interesting to examine whether the other lipid biosynthetic enzymes could sense the levels of downstream lipid metabolites and be allosterically regulated.

## MATERIALS AND METHODS

### Protein expression and purification

The AtSPT and AtSPT-ORM1 complexes were recombinantly expressed and purified similarly as previously described (*25*). In brief, the codon-optimized cDNAs for LCB1 (UniProt: Q94IB8), LCB2a (UniProt: Q9LSZ9), and ORM1 (UniProt: Q9C5I0) were individually subcloned into the pCAG vector without a tag, whereas the codon-optimized cDNA for SPTssa (UniProt: A8MSB8) was subcloned into the same vector with an N-terminal Flag tag. A list of primers used in this study is provided in table S3. Each liter of HEK293F cells at a cell density of 2.5 to 3.0 × 10^6^ cells per ml in SMM 293T-II medium (Sino Biological Inc.) was transiently cotransfected with 2 mg of plasmid mixtures (LCB1:LCB2a:SPTssa = 3:3:1 or LCB1:LCB2a:ORM1:SPTssa = 3:3:3:1 by weight) and 6 mg of polyethyleneimine (YEASEN). After transfection at 37°C for 12 hours, 10 mM sodium butyrate was supplied to improve protein expression for 48 hours. The cells were harvested and resuspended in buffer containing 25 mM Hepes (pH 7.5), 150 mM NaCl, and a protease inhibitor cocktail (Sigma-Aldrich). After membrane solubilization with 1% (w/v) GDN (Anatrace) at 4°C for 2 hours, followed by centrifugation at 20,000*g* for 1 hour, the supernatant was collected and purified with anti-Flag G1 affinity resin (GenScript). The resin was washed with buffer A [25 mM Hepes (pH 7.5), 150 mM NaCl, and 0.01% GDN] plus 5 mM adenosine 5′-triphosphate–MgCl_2_ and eluted with buffer A supplied with Flag peptide (200 μg/ml; GenScript). The concentrated eluent was applied to Superose 6 10/300 GL column (GE Healthcare) equilibrated with buffer A. The peak protein fractions from size exclusion chromatography were pooled and concentrated for further biochemical or cryo-EM studies.

### SPT activity assay

SPT activity was determined by a continuous spectrophotometric assay detecting the release of CoA-SH from palmitoyl-CoA similarly as previously reported (*25*, *44*). CoA-SH reacts with 5,5′-dithiobis-2-nitrobenzoic acid (DTNB) to form the yellow-colored TNB (5-thio-2-nitrobenzoic acid), which exhibits maximum absorbance at 412 nm. A standard curve with CoA-SH concentrations varying between 10 and 250 μM was generated. The assays were carried out on a 50-μl scale at 30°C in 96-well plates with a BioTek plate reader. Measurements were taken at 412 nm for 1 hour following reaction initiation. A typical assay setup at saturating conditions contained 0.3 μM SPT or SPT-ORM1, 25 μM PLP, 0.4 mM DTNB, 100 mM l-serine, and 100 μM palmitoyl-CoA in buffer A. To measure the curve of SPT activity versus various concentrations of l-serine, the palmitoyl-CoA concentration was maintained at 100 μM. To measure the curve of SPT activity versus various concentrations of palmitoyl-CoA, the l-serine concentration was maintained at 100 mM. The SPT activity was determined from the raw data that falls in the linear range and calculated on the basis of the standard curve. The statistical analysis was conducted with GraphPad Prism 8. For all curves and dot plot graphs, each data point is the average of three independent experiments, and error bars stand for SEM. The curve of SPT activity versus l-serine concentration follows the Michaelis-Menten equation. The curve of SPT activity versus palmitoyl-CoA concentration follows an allosteric sigmoidal equation.

C6-ceramide (Avanti, 860506P), C6-phytoceramide (GlpBio, GC43104), and C6-dihydroceramide (Cayman, 24368) were dissolved in dimethyl sulfoxide (DMSO) at concentration ranges from 0.18 μM to 5 mM as 100× stocks. To measure the SPT activity inhibition curves of SPT or SPT-ORM1 complexes by ceramide analogs, each protein complex at a concentration of 3 μM was incubated with various concentrations of 10× ceramide analogs on ice for 1 hour before being diluted 10 times for reaction initiation. The SPT activity was normalized relative to that of the SPT or SPT-ORM1 complexes at a very low concentration of ceramide analogs. The SPT activity inhibition curves follow the nonlinear regression equation: *Y* = bottom + (1 − bottom)/[1 + (*X*/IC_50_)]. All the IC_50_ values were summarized in table S2. The SPT-specific inhibitor myriocin was dissolved in DMSO at 1 mM as a 100× stock. The activity of SPT or SPT-ORM1 in the presence of 10 μM myriocin was measured similarly.

### MS and TLC analysis

The LC-MS/MS was performed similarly as previously reported (*45*, *46*). Briefly, chloroform and methanol were mixed with 1 ml of SPT-ORM1 (0.3 mg in total) with a volume ratio of 0.5:1:1 for lipid extraction. After sonication and centrifugation, the lower chloroform phase was collected and dried under a nitrogen stream. The lipids were dissolved in 30 μl of acetonitrile (ACN)/isopropyl alcohol (IPA)/H_2_O (65:30:5, v/v/v) solution with 5 mM ammonium acetate. About 5 μl of sample was injected into a C18 reversed-phase column (1.9 μm, 2.1 mm by 100 mm; Thermo Fisher Scientific) in the LC-MS/MS system (Q Exactive Orbitrap Mass Spectrometer, Thermo Fisher Scientific). Mobile phases A and B were ACN/H_2_O (60:40, v/v) with 10 mM ammonium acetate and IPA/ACN (90:10, v/v) with 10 mM ammonium acetate, respectively. The elution gradient was set between 32 and 85% B. The MS was performed in negative mode with a spray voltage of 3.5 kV. Full-scan MS spectra were obtained over the mass/charge ratio (*m*/*z*) range of 166.7 to 2000. The MS/MS spectra were searched across the lipid database by LipidSearch software (Thermo Fisher Scientific). The peaks for ceramide ions from the *m*/*z* range of 550 to 710 were presented. For TLC, the lipids extracted similarly were resuspended in 10 μl of chloroform and subjected to a TLC silica gel 60 F_254_ plate (Merck). The plate was developed using a chloroform-methanol (190:10 v/v) solvent through two consecutive runs in the same direction. The lipids were visualized by iodine vapor staining of the TLC plate overnight.

### Cryo-EM sample preparation and data collection

Quantifoil Cu R1.2/1.3 300 mesh grids were glow-discharged in a PELCO easiGlow device at 15 mA for 45 s before use. Three microliters of purified SPT or SPT-ORM1 (WT, ORM1-N17A, and LCB2a-ΔN5) complexes at ~18 mg/ml were applied to the glow-discharged grids. For SPT and SPT-ORM1 (LCB2a-ΔN5) complexes, the purified protein was preincubated with 0.5 mM PLP, 10 mM l-serine, and 1 mM S-CoA on ice for 1 hour before sample preparation. The grids were blotted for 4.5 s and flash-frozen in liquid ethane cooled by liquid nitrogen using Vitrobot (Mark IV, Thermo Fisher Scientific) at 8°C with 100% humidity. Images were collected using SerialEM (*47*) on a Titan Krios microscope operating at 300 kV, equipped with a K2 Summit camera (Gatan) and a Quantum energy filter (Gatan) with a slit width of 20 eV. The micrographs were collected at a nominal magnification of ×130,000 in superresolution mode with a calibrated pixel size of 0.54 Å. Each stack was exposed for 5.76 s in 32 frames with a total dose of 50 e^−^/Å^2^. Defocus values ranged from −2.0 to −1.0 μm. The stacks were motion-corrected and binned twofold using MotionCor2 (*48*) with dose weighting (*49*), resulting in a pixel size of 1.08 Å. The defocus values were estimated with Gctf (*50*).

### Cryo-EM data processing

The data processing was mainly performed in Relion 3.0 (*51*). For the WT SPT-ORM1 complex dataset, a total of 801,707 particles were automatically picked from 1849 micrographs. The 2D classification generated 723,325 good particles that were used for a global search 3D classification. A total of 426,406 particles were selected and used for 3D autorefinement. The particles were used for further 3D classifications with or without a monomer mask. The local search 3D classification without mask generated a good 3D class with 159,298 particles. 3D autorefinement of the particles with overall mask and C2 symmetry yielded a reconstruction with an overall resolution of 3.2 Å. The 3D classification with monomer mask generated 115,440 particles with the best quality. 3D autorefinement of the particles with a monomer mask resulted in an EM map at 3.2 Å.

The data processing procedures for SPT-ORM1 (ORM1-N17A) and SPT-ORM1 (LCB2a-ΔN5) were similar as above. In brief, 930,704 and 893,978 particles were picked from 1768 and 1675 micrographs of SPT-ORM1 (ORM1-N17A) and SPT-ORM1 (LCB2a-ΔN5), respectively. 2D classifications resulted in 538,975 and 721,401 good particles that were subjected to global search 3D classifications and autorefinements. Further 3D classifications with monomer masks generated a good 3D class for SPT-ORM1 (ORM1-N17A) and SPT-ORM1 (LCB2a-ΔN5) at resolutions of 3.7 and 2.8 Å, respectively. Further nonuniform refinement and local refinement of the SPT-ORM1 (ORM1-N17A) particles using cryoSPARC (*52*) improved the resolution to 3.4 Å. Resolutions were estimated by the gold-standard Fourier shell correlation 0.143 criteria (*53*) with high-resolution noise substitution (*54*).

### Model building and refinement

The human SPT-ORMDL3* complex structure (PDB 6M4O) was used as the initial model for WT SPT-ORM1 model building. The model was docked into the EM map with Chimera, and each residue was manually fitted in Coot (*55*). Structural refinements were performed using Phenix (*56*) in real space with secondary structure and geometry restraints. The WT SPT-ORM1 structure was used as the initial model for the model building of SPT-ORM1 (ORM1-N17A) and SPT-ORM1 (LCB2a-ΔN5). All the structural models were validated with Phenix and MolProbity (*57*). The model refinement and validation statistics were summarized in table S1.

### Functional characterization of AtORM1 mutations in HEK293 *SPTSSA* KO cells

HEK293 *SPTSSA* KO cells were generated using a kit (KN409747, OriGene). Briefly, transfected HEK cells were passaged seven times before applying puromycin selection. Single colonies were isolated and propagated for identification of deletion clones by quantitative polymerase chain reaction. Homozygous KO lines were verified by sequencing and immunoblotting. Plasmids expressing AtLCB1, AtLCB2a, and Flag-AtSPTssa were transfected into HEK293 *SPTSSA* KO cells along with 0, 75, 150, or 300 ng of an AtORM1-expressing plasmid. When deuterium-labeled serine (3,3-d2 l-serine, 3 mM; DLM-161, Cambridge Isotope Laboratories) was used, it was added 24 hours before harvesting. Cells were harvested; washed in phosphate-buffered saline, suspended in 50 mM tris (pH 7.5), 1 mM EGTA, and 15% glycerol with protease and phosphatase inhibitors; and split for lipid extraction and protein quantification.

Cells were lysed by sonication in 25 mM tris (pH 7.5), 50 mM NaCl, 1 mM EGTA, 10% glycerol, 0.3% NP-40, 0.3% deoxycholate, and 0.03% SDS with protease and phosphatase inhibitors (cOmplete protease inhibitor and PhosSTOP phosphatase cocktails, Sigma-Aldrich). Protein concentrations were determined by Bio-Rad assay. Proteins were resolved by electrophoresis on NuPAGE Novex 4 to 12% Bis-Tris gels (Thermo Fisher Scientific) and transferred to nitrocellulose. Membranes were incubated in intercept blocking buffer for 1 hour, incubated with primary antibody overnight, and washed and incubated with IRDye 800CW goat anti-rabbit (LI-COR Biosciences), and bands were visualized using the Odyssey System (LI-COR) and Image Studio (LI-COR). The AtLCB1 antibody was generated by Vivitide (Gardner, MA) using two peptides (amino acids 79 to 92 EPLIPPITEDMKHE and 459 to 473 HSESDLLKASESLKR). The AtLCB2a antibody was raised in rabbits (Cocalico Biologicals, Stevens, PA) against *Escherichia coli*–generated protein encompassing the C-terminal 166 amino acids of the AtLCB2a. The AtORM1 antibody was generated by Labcorp (Denver, PA) using two peptides (amino acids 72 to 85 KGTPFADDQGIYNG and 90 to 103 EQMDNGQQLTRNRK). Anti-Flag antibody was from OriGene.

MS analysis was conducted as previously described (*58*). In brief, cell pellets (0.1 to 0.2 mg) were added to 1 ml of methanol containing 25 pmol of internal standards (Avanti Polar Lipids, LM6002) and bath sonicated for several minutes. Following addition of 0.5 ml chloroform, samples were incubated overnight at 48°C, briefly centrifuged to remove insoluble material, and dried under nitrogen. The mass spectrometer was set to detect compounds in MRM mode. Compounds were quantified by taking the ratio of the peak area to the known concentration of the representative internal standard using the AB Sciex Analyst program and normalized to protein concentration.

## Notes

View/request a protocol for this paper from *Bio-protocol*.
