# Supplementary material for: Mechanism of sphingolipid homeostasis revealed by structural analysis of Arabidopsis SPT-ORM1 complex
Source: Sci Adv. 2023 Mar 29;9(13):eadg0728. doi: 10.1126/sciadv.adg0728 (PMC10058238; doi:10.1126/sciadv.adg0728)
Supplement: Supplementary file 1 — Figs. S1 to S15 Tables S1 to S3 [file sciadv.adg0728_sm.pdf]

Science Advances
AAAS

Supplementary Materials for

Mechanism of sphingolipid homeostasis revealed by structural analysis of *Arabidopsis* SPT-ORM1 complex

Peng Liu *et al.*

Corresponding author: Xin Gong, gongx@sustech.edu.cn

Sci. Adv. **9**, eadg0728 (2023)
DOI: 10.1126/sciadv.adg0728

This PDF file includes:

Figs. S1 to S15
Tables S1 to S3

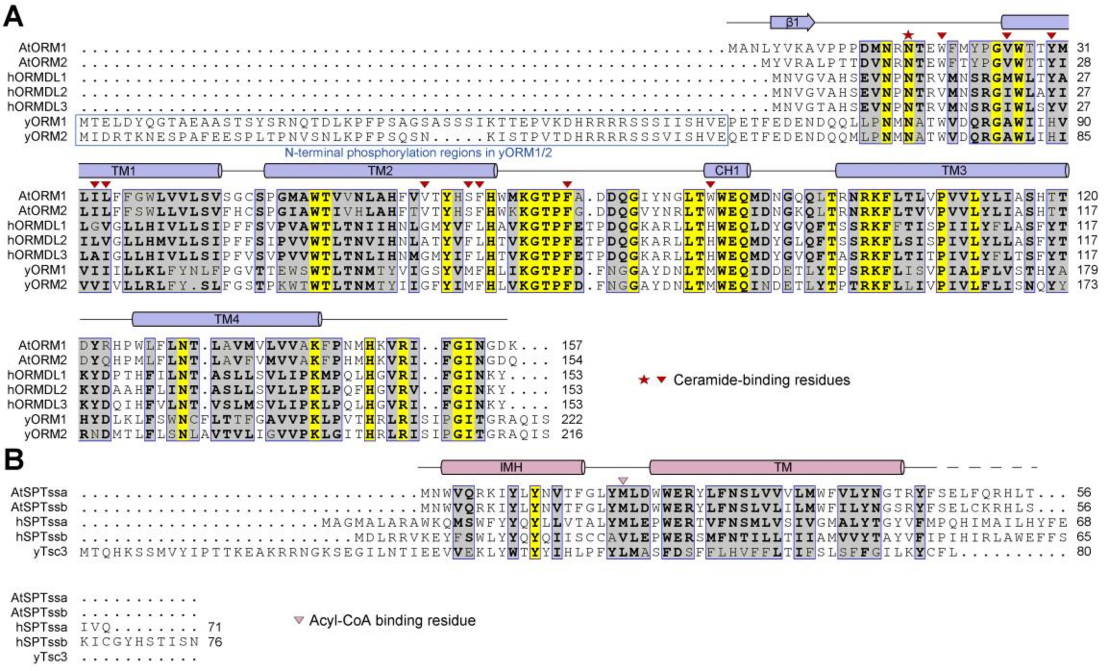

Fig. S1 | Sequence alignments of ORMs/ORMDLs (A) and small regulatory subunits (B) from different species. Secondary structural elements of *Arabidopsis thaliana* ORM1 and SPTssa are labeled above the sequence alignments. TM: transmembrane helix. CH: cytosolic helix. IMH: in-plane membrane helix. The red star shows the invariant asparagine residue that contributes to the coordination of the polar head of ceramide. The red triangles show the remaining residues for ceramide coordination in *Arabidopsis* ORM1. The pink triangle indicates the conserved residue responsible for acyl-CoA selectivity in the small regulatory subunits. The UniProt IDs for the aligned ORM/ORMDL sequences are as follows: AtORM1: Q9C5I0; AtORM2: Q9FHY3; hORMDL1: Q9P0S3; hORMDL2: Q53FV1; hORMDL3: Q8N138; yORM1: P53224; and yORM2: Q06144. The UniProt IDs for the aligned small regulatory subunit sequences are as follows: AtSPTssa: A8MSB8; AtSPTssb: F4IPU5; hSPTssa: Q969W0; hSPTssb: Q8NFR3; yTsc3: Q3E790. “At” for *Arabidopsis thaliana*, “h” for *Homo sapiens* (Human), and “y” for *Saccharomyces cerevisiae* (yeast).

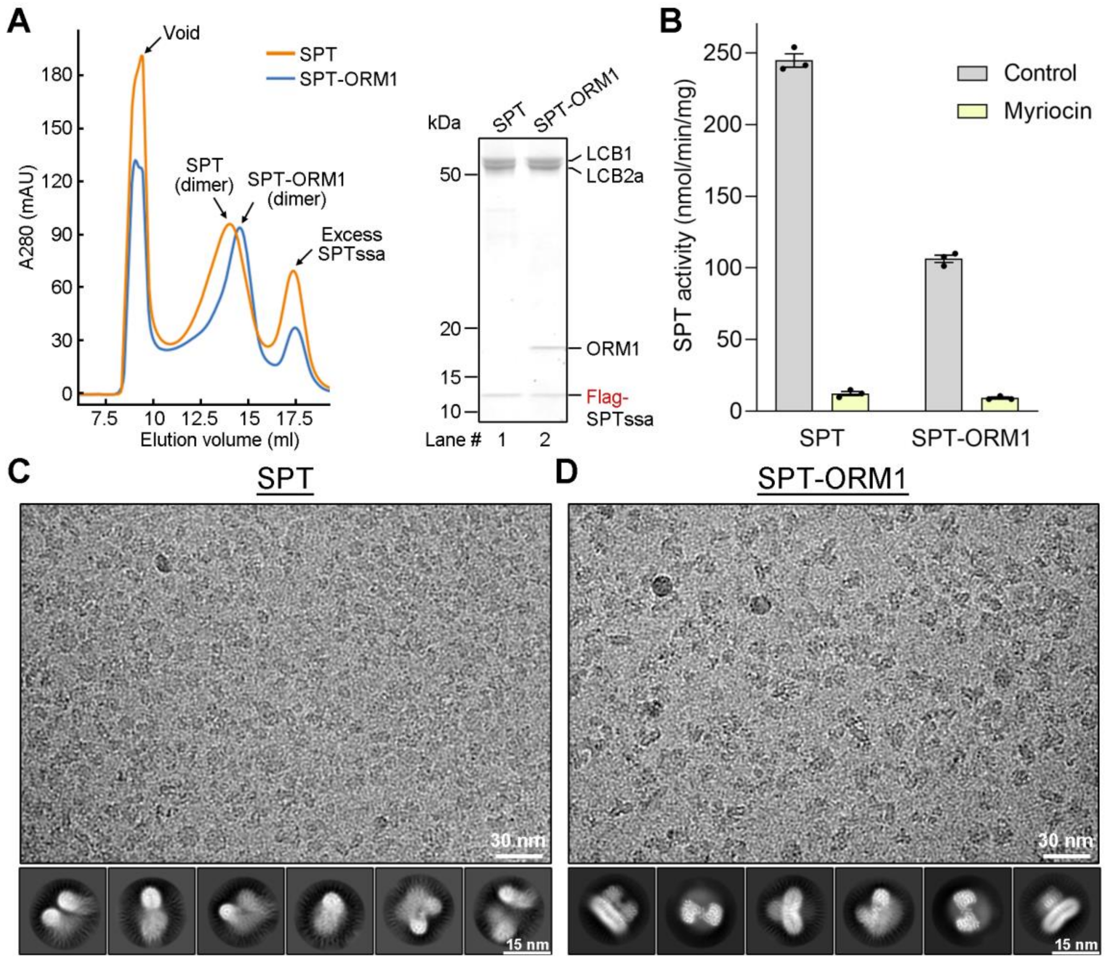

Fig. S2 | Biochemical characterization and cryo-EM study of the *Arabidopsis* SPT and SPT-ORM1 complexes. **A,** Size exclusion chromatography (SEC) profiles and coomassie blue-stained SDS-PAGE gel of the SPT and SPT-ORM1 complexes. **B,** SPT activity of purified SPT and SPT-ORM1 complexes. Both complexes could be inhibited by myriocin. **C-D,** Representative cryo-EM micrograph and 2D class averages of the SPT (**C**) and SPT-ORM1 (**D**) complexes.

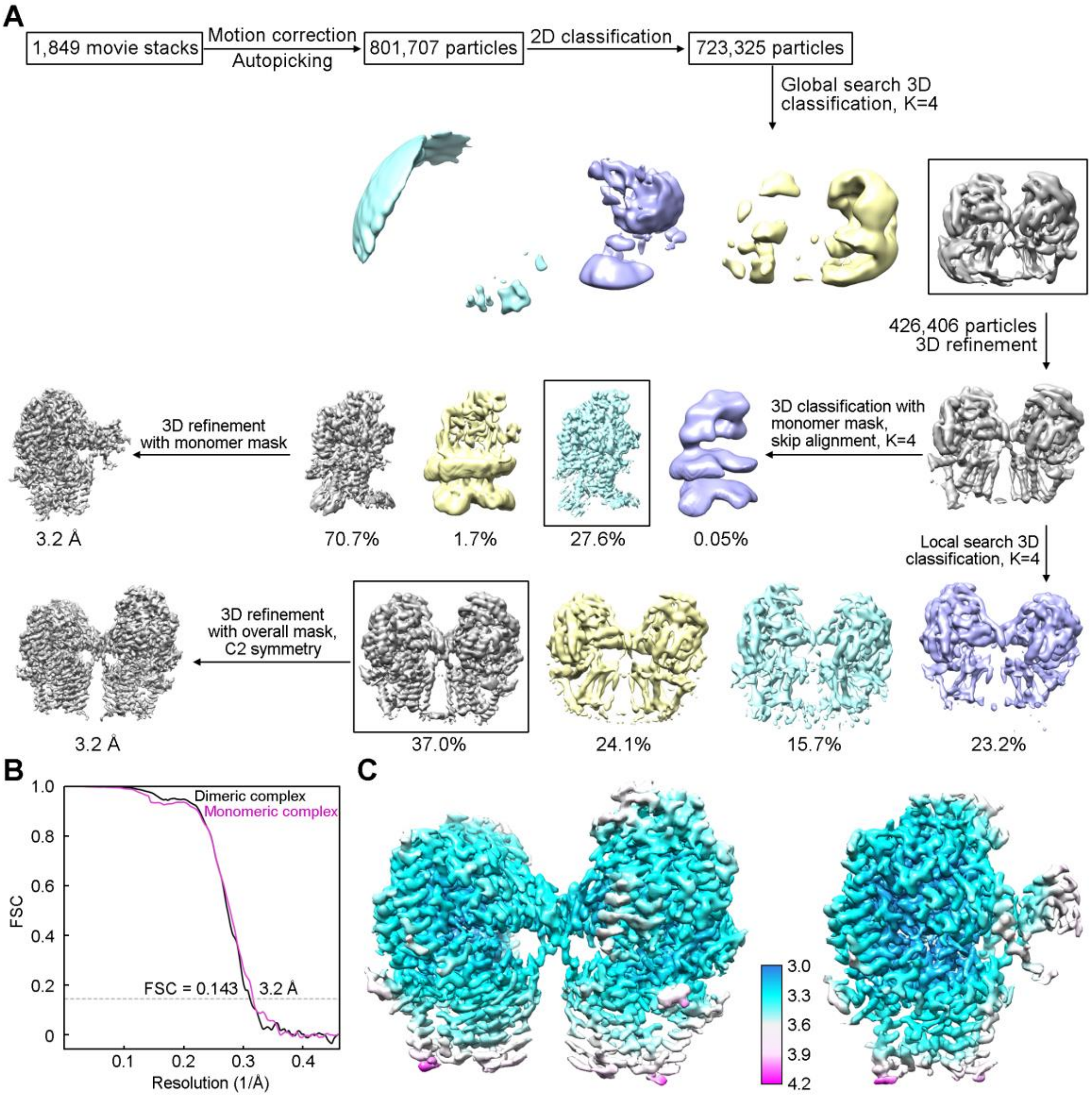

Fig. S3 | Cryo-EM analysis of the SPT-ORM1 complex. **A,** Flowchart for cryo-EM data processing. **B,** Gold-standard FSC curves for SPT-ORM1 dimeric and monomeric maps generated using Relion 3.0. **C,** Local resolution maps of the dimeric and monomeric SPT-ORM1. The color code for resolutions, shown with the unit Å, is calculated using Relion 3.0.

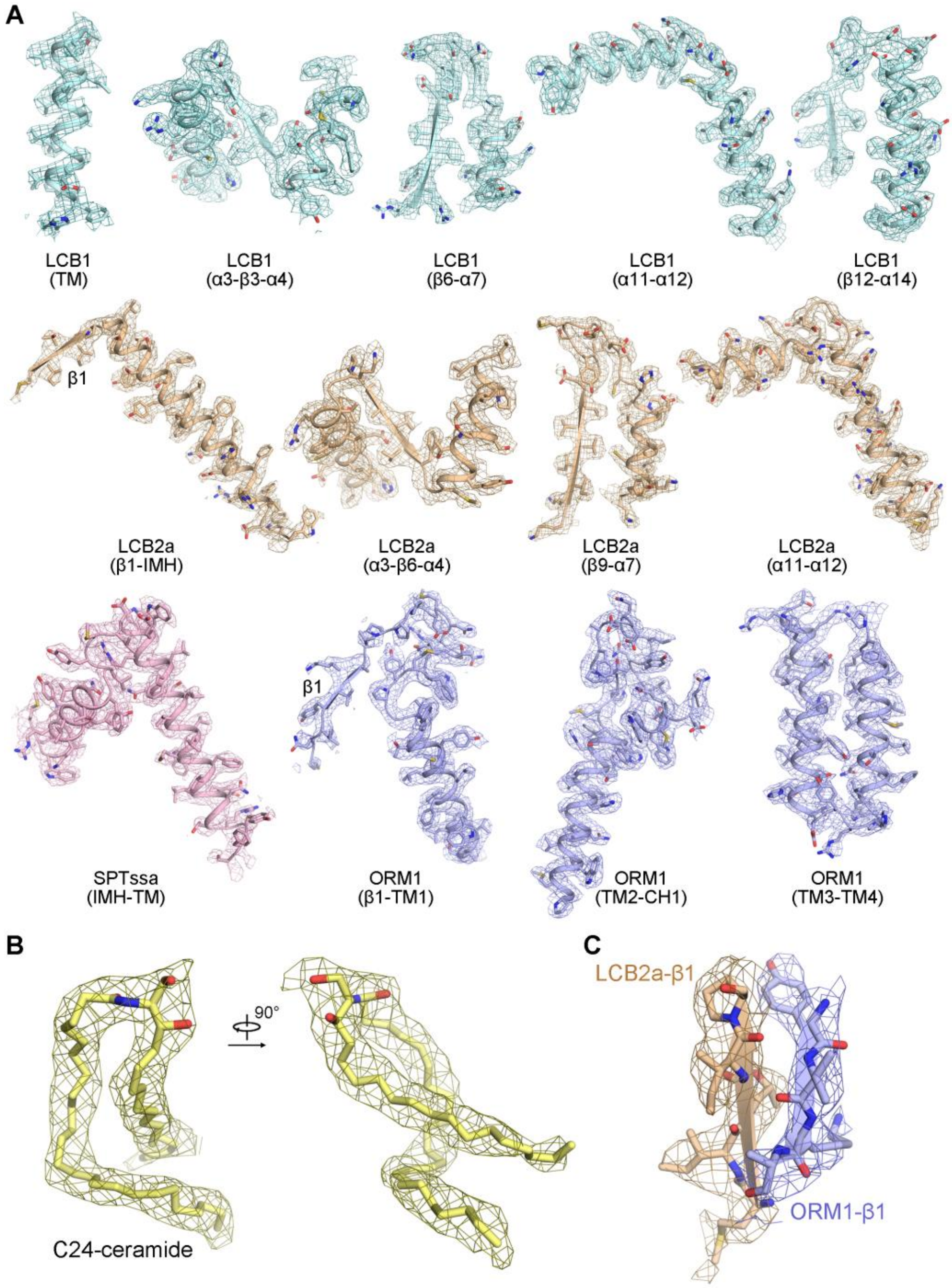

Fig. S4 | Representative EM maps of the SPT-ORM1 complex. **A,** EM maps of the representative secondary structural elements from SPT-ORM1 complex. **B,** Close-up views of the EM map for ceramide. **C,** A close-up view of the EM map for the hybrid β-sheet between the N-termini of ORM1 and LCB2a. The densities, shown in meshes, were contoured at 5σ.

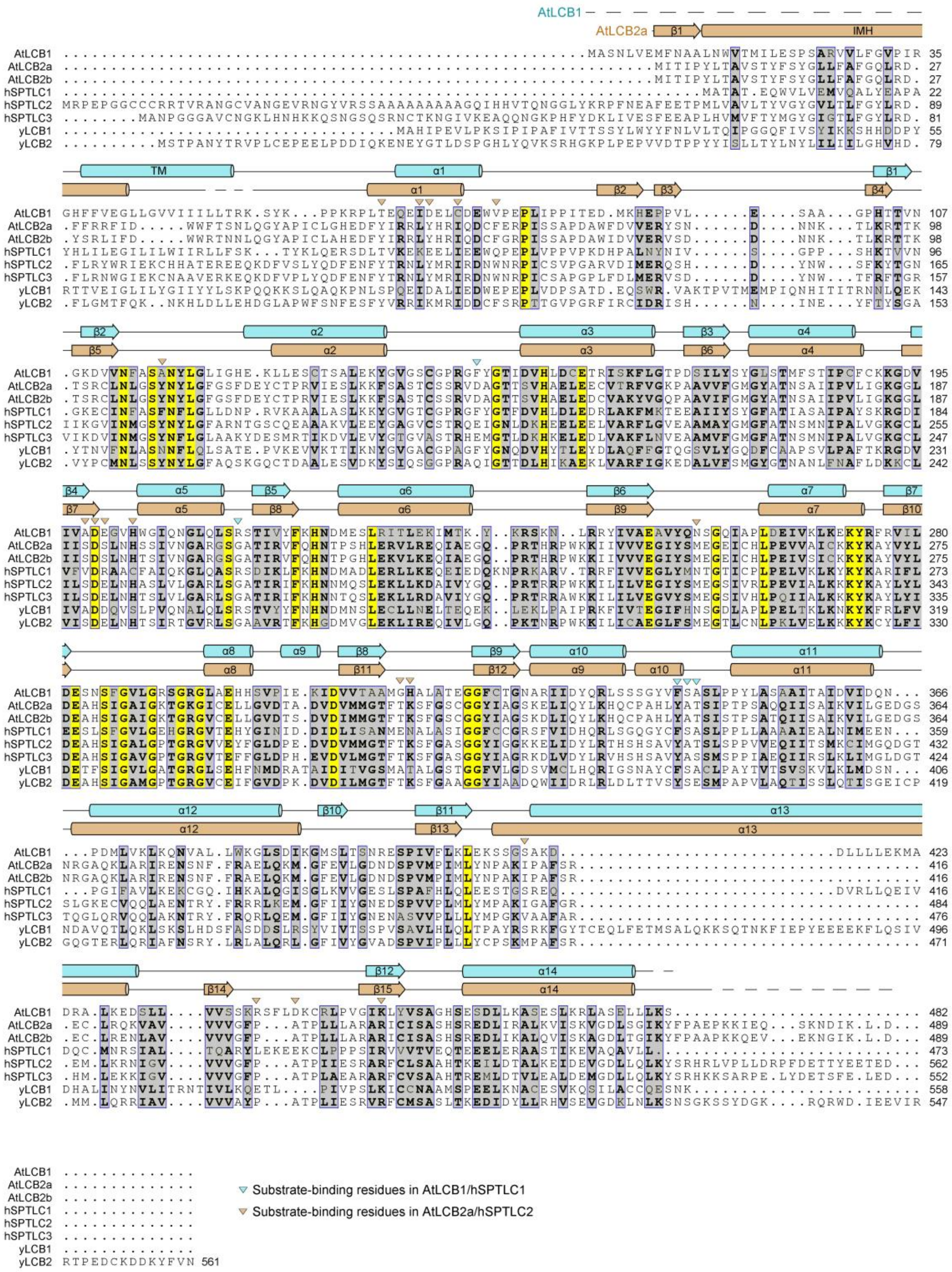

Fig. S5 | Sequence alignments of LCB/SPTLC subunits from different species. Secondary structural elements of *Arabidopsis thaliana* LCB1 and LCB2a are labeled above the sequence alignments. The cyan triangles indicate the conserved substrate-binding residues in AtLCB1 and hSPTLC1; the wheat triangles show the conserved

substrate-binding residues in AtLCB2a and hSPTLC2. The UniProt IDs for the aligned LCB/SPTLC sequences are as follows: AtLCB1: Q94IB8; AtLCB2a: Q9LSZ9; AtLCB2b: Q9M304; hSPTLC1: O15269; hSPTLC2: O15270; hSPTLC3: Q9NUV7; yLCB1: P25045; yLCB2: P40970.

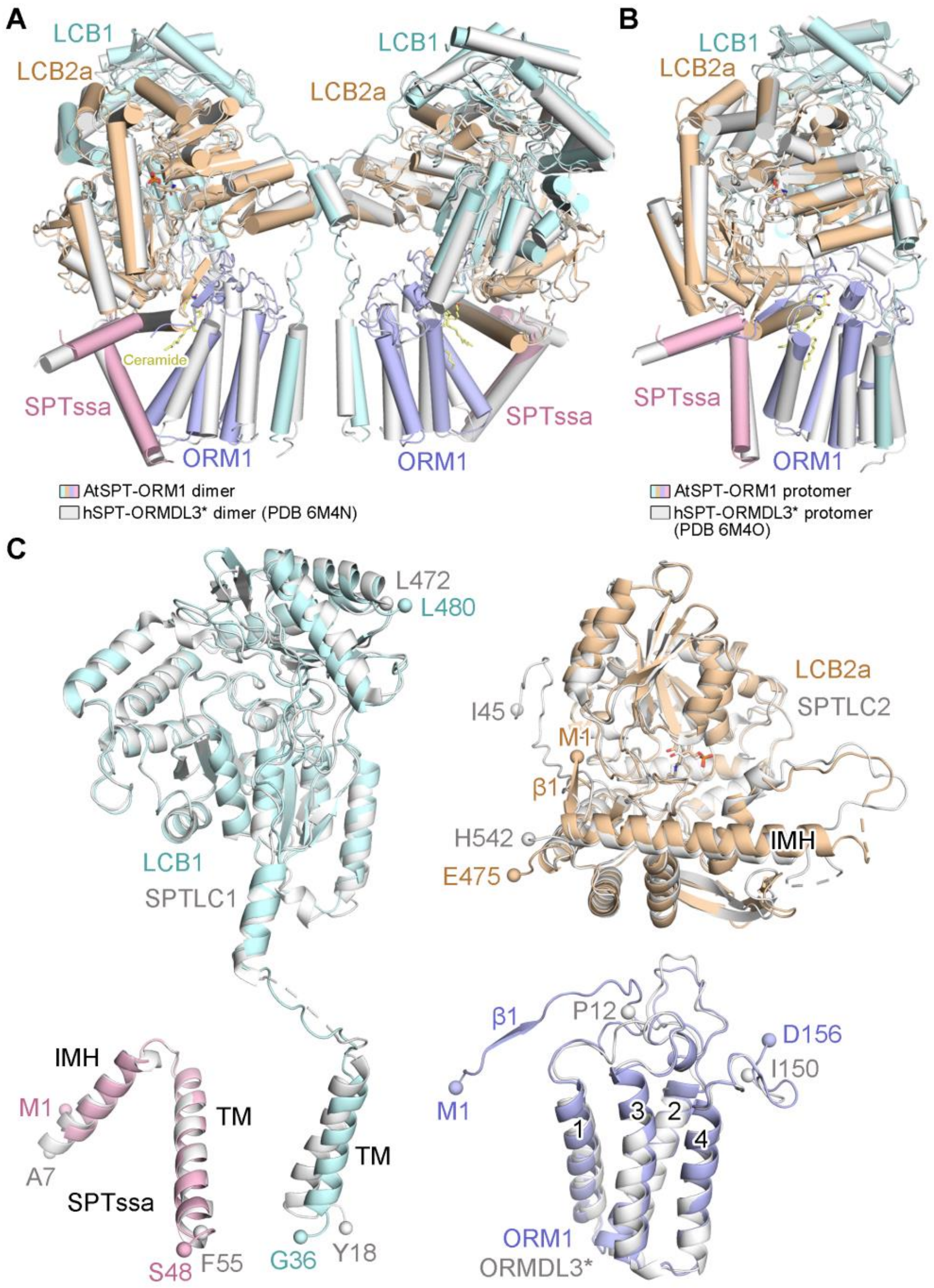

Fig. S6 | Structural comparison of AtSPT-ORM1 and hSPT-ORMDL3. A-B, Structural comparison of the dimeric (**A**) and monomeric (**B**) AtSPT-ORM1 (colored based on the subunits) and hSPT-ORMDL3* (gray, PDB 6M4N and 6M4O) complexes. **C,** Structural comparisons of the four individual subunits between AtSPT-ORM1 and hSPT-ORMDL3*. The resolved N- and C-termini of each subunit are shown in spheres.

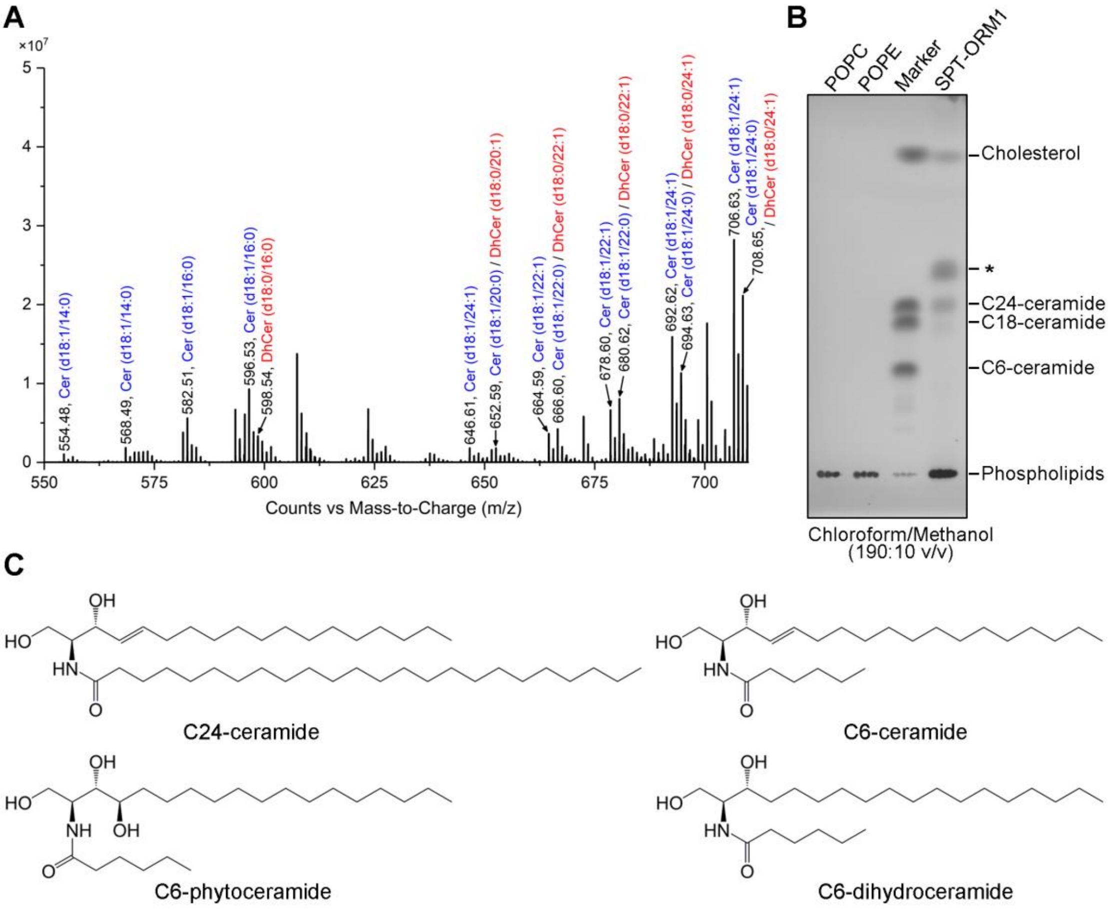

Fig. S7 | Characterization of the co-purified ceramides in SPT-ORM1 complex and chemical structure of the natural C24-ceramide and the short-chain ceramide analogs used in this study. A, Mass spectrometry analysis of ceramides co-purified with SPT-ORM1. The major ceramide (Cer) and dihydroceramide (DhCer) species were indicated in the mass spectra data. All the ions were confirmed by tandem MS spectra. **B,** Visualization of the endogenous ceramides co-purified with SPT-ORM1 by TLC. The asterisk marks an unidentified component. **C,** Chemical structure of C24-ceramide, C6-ceramide, C6-phytoceramide, and C6-dihydroceramide.

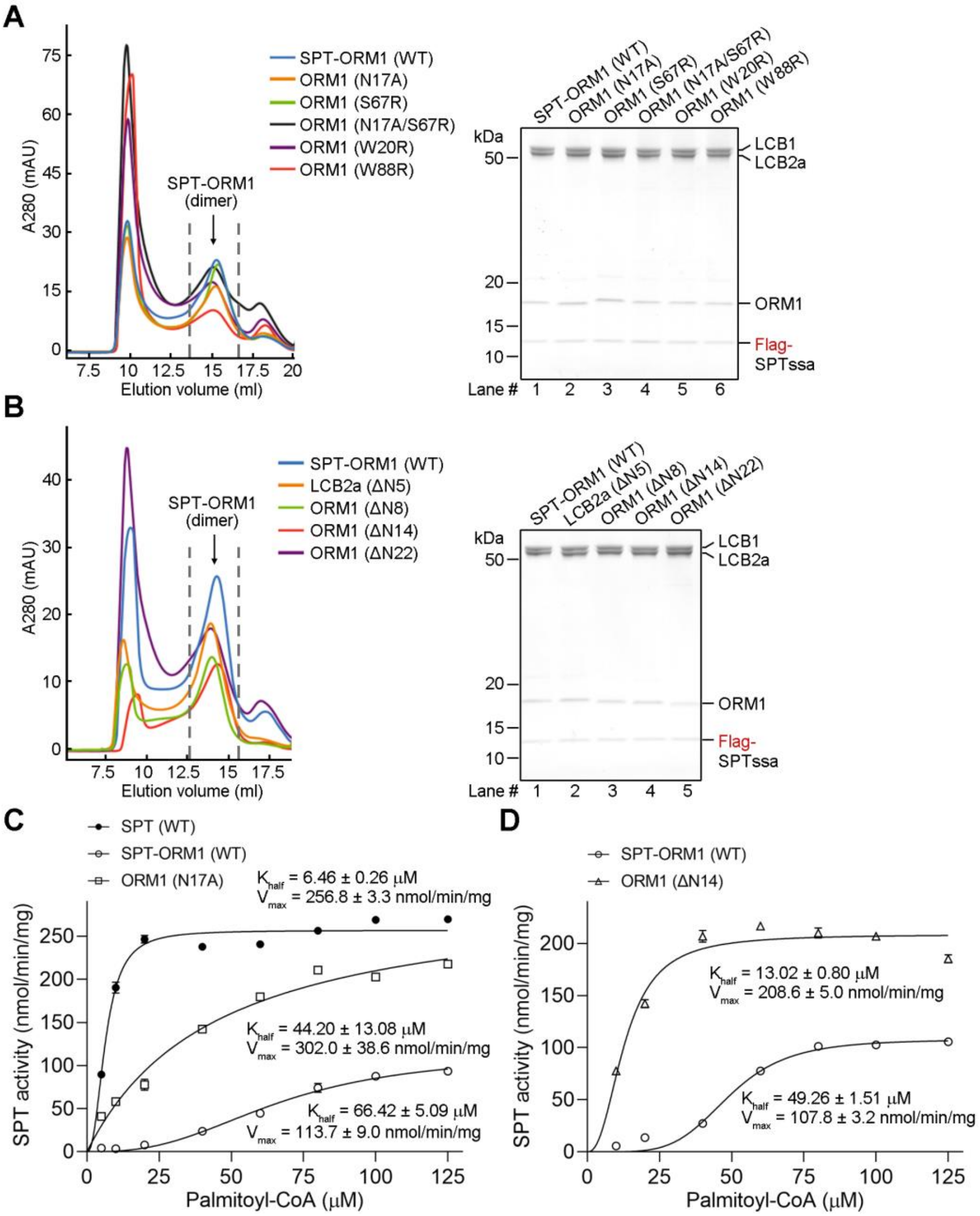

Fig. S8 | SEC profiles and enzymatic kinetics of SPT-ORM1 variants. A-B, SEC profiles of the ceramide-binding mutants (**A**) and the N-terminal deletion of LCB2a or ORM1 (**B**). Protein fractions of the dimeric SPT-ORM1 variants were collected for biochemical studies. The protein was visualized by Coomassie-blue stained SDS-PAGE gel. **C,** Enzymatic kinetics of the ORM1-N17A variant compared to those of the WT SPT and SPT-ORM1 complexes. **D,** Enzymatic kinetics of the ORM1-ΔN14 variant compared to those of the WT SPT-ORM1 complex.

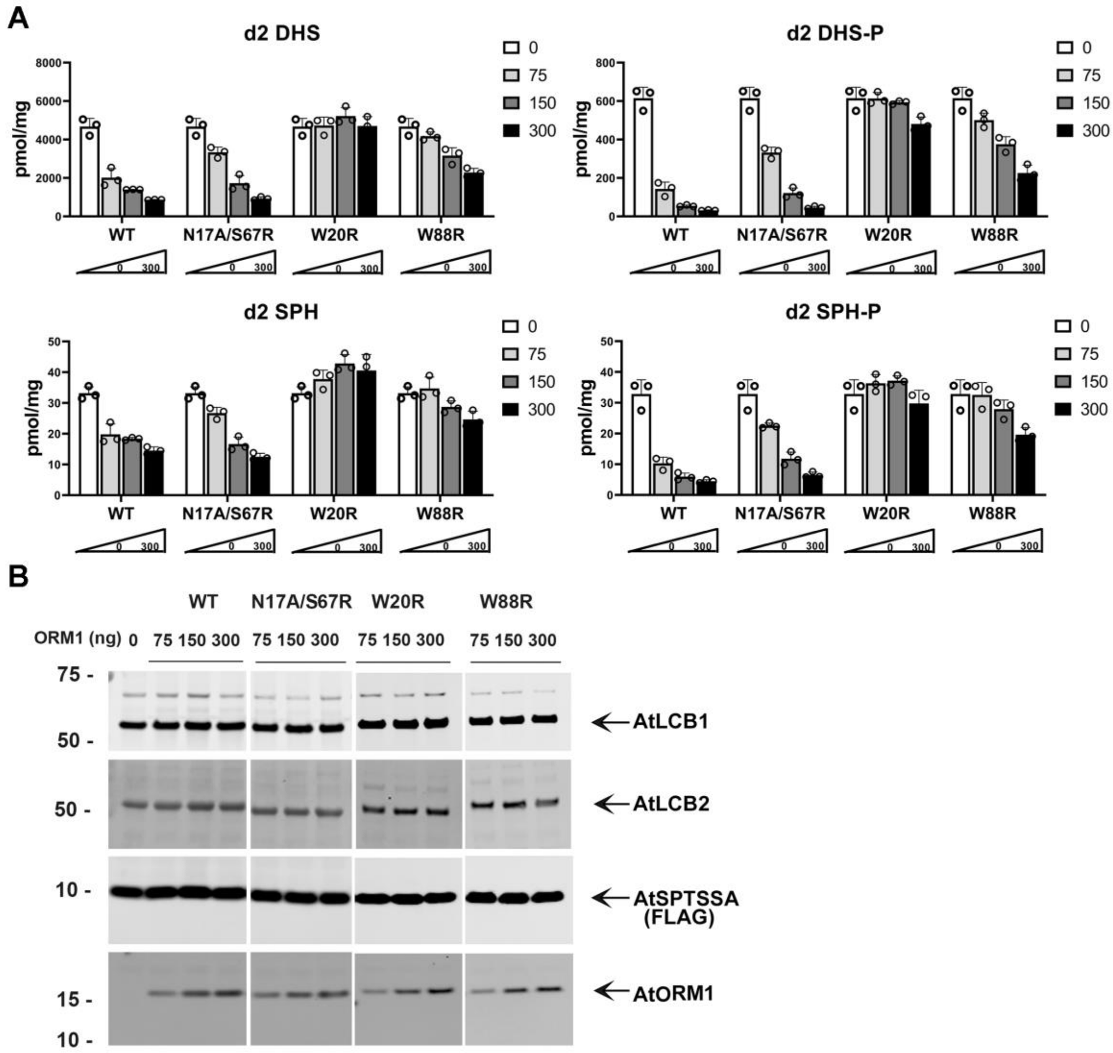

Fig. S9 | The ceramide binding site mutations in AtORM1 interfere with ORM1-mediated repression of SPT. Plasmids expressing AtLCB1, AtLCB2a, and Flag-AtSPTssa were transfected into HEK293 SPTSSA knockout cells along with increasing amounts (0, 75, 150, or 300 ng) of WT AtORM1 or point mutation (N17A/S67R, W20R, or W88R) plasmids. After 16 hours, d2-serine was added, and 24 hours later cells were harvested and deuterated LCBs (DHS, DHS-P; SPH, SPH-P) were quantitated by LC-MS (**A**). The microsomal proteins from the cells above were separated by SDS-PAGE and the indicated proteins were analyzed by immunoblotting (**B**). DHS = dihydrosphingosine; DHS-P = DHS-1-phosphate; SPH = sphingosine; SPH-P = SPH-1-phosphate.

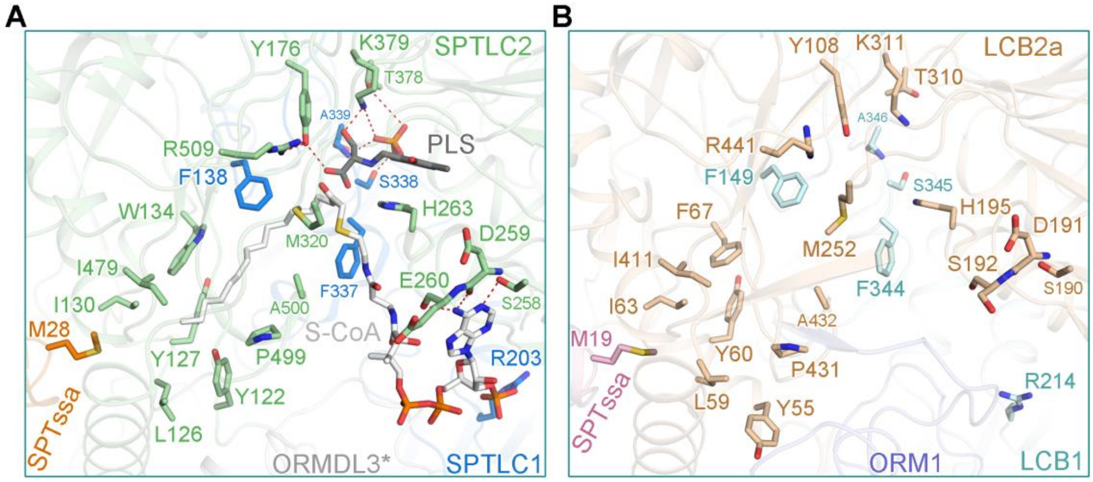

Fig. S10 | The conserved substrate-binding site in hSPT-ORMDL3 (A) and AtSPT-ORM1 (B). The substrate-binding residues in PLS- and S-CoA-bound hSPT-ORMDL3* (PDB 7CQK) are shown as sticks and the potential polar interactions with substrates are displayed by red dashed lines (**A**). The corresponding substrate-binding residues in AtSPT-ORM1 are shown as sticks (**B**).

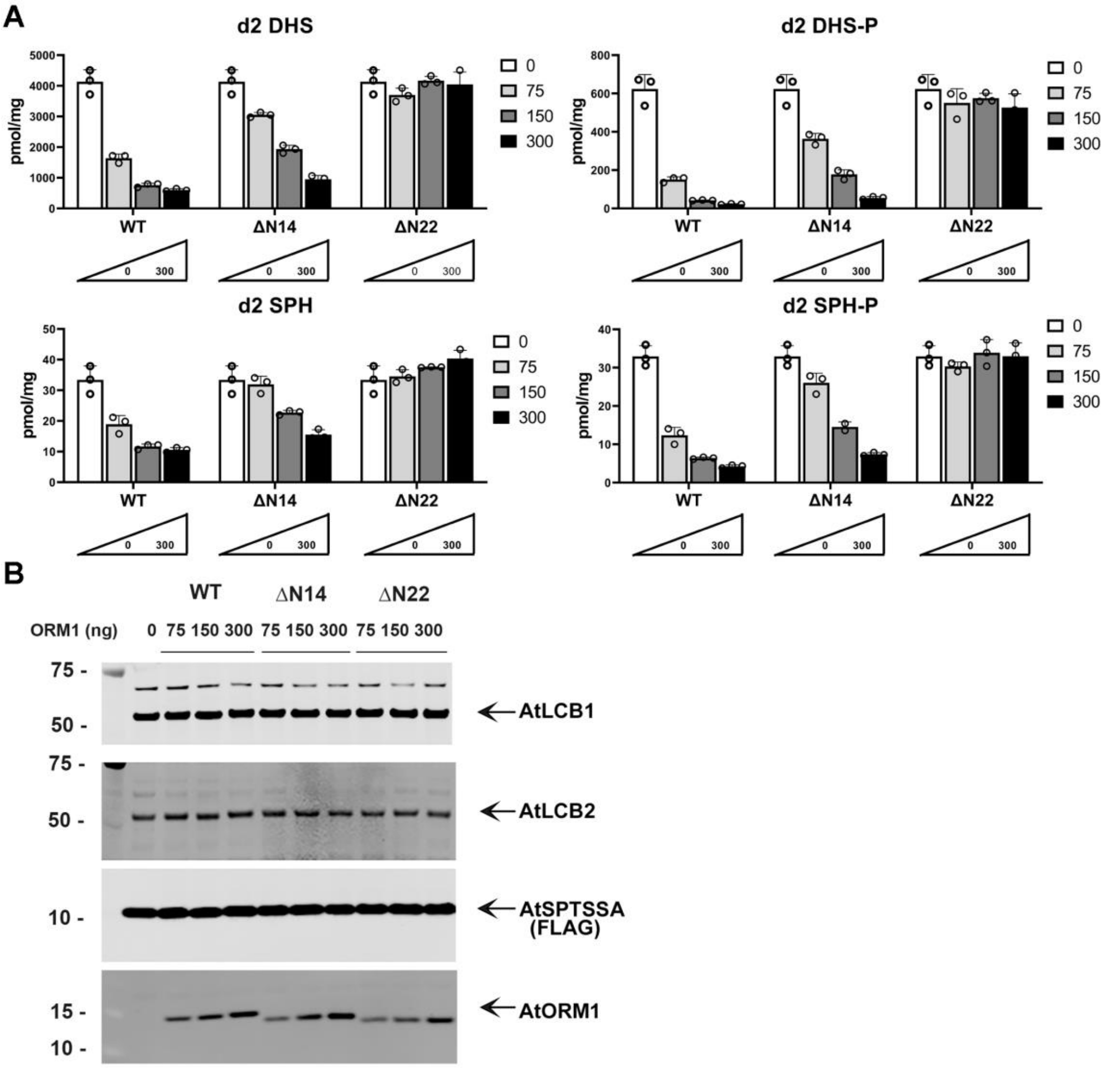

Fig. S11 | The AtORM1 N-terminal deletions impair ORM1-mediated inhibition of SPT. Plasmids expressing AtLCB1, AtLCB2a, and Flag-AtSPTssa were transfected into HEK293 SPTSSA knockout cells along with increasing amounts (0, 75, 150, or 300 ng) of WT AtORM1 or variants with N-terminal deletions (ΔN14 or ΔN22). After 16 hours, d2-serine was added, and 24 hours later cells were harvested and deuterated LCBs (DHS, DHS-P; SPH, SPH-P) were quantitated by LC-MS (**A**). The microsomal proteins from the cells above were separated by SDS-PAGE and the indicated proteins were analyzed by immunoblotting (**B**).

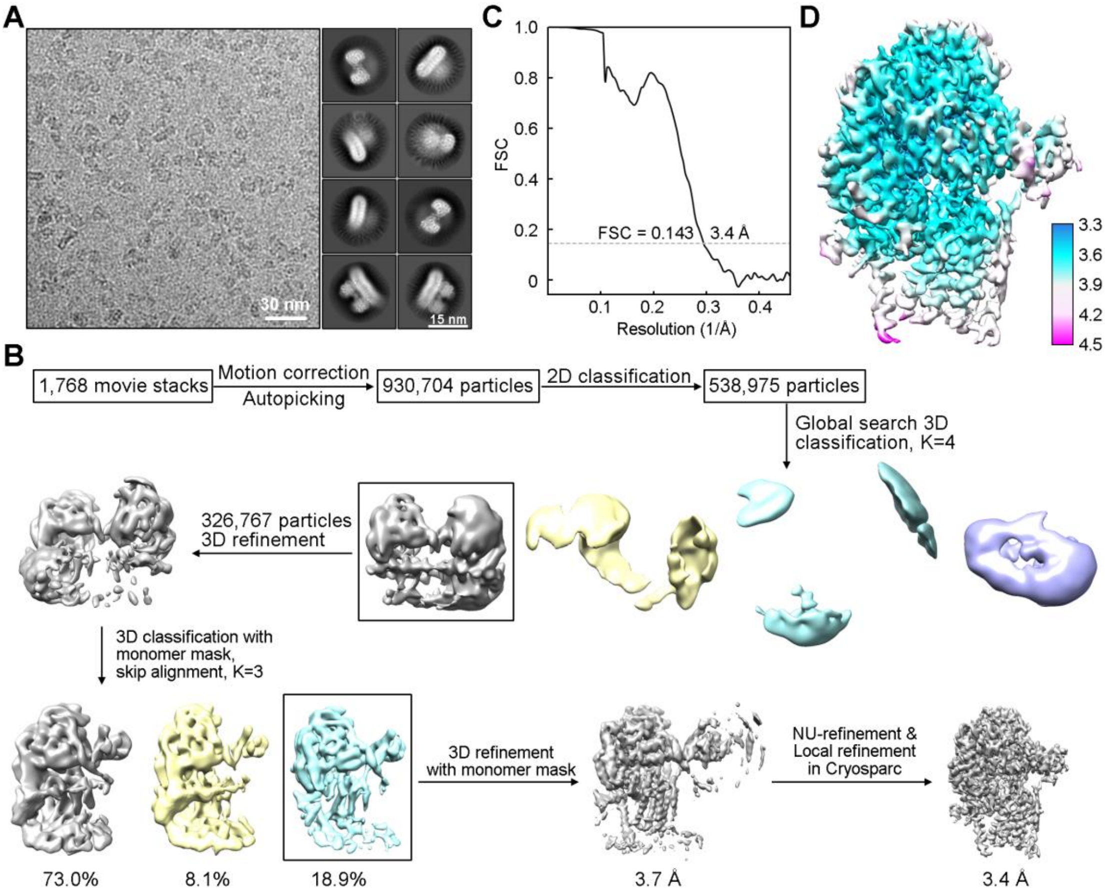

Fig. S12 | Cryo-EM analysis of the SPT-ORM1 (ORM1-N17A) complex. **A,** Representative cryo-EM micrograph and 2D class averages. **B,** Flowchart for cryo-EM data processing. **C,** Gold-standard FSC curve for ORM1-N17A mutant map generated using Relion 3.0. **D,** Local resolution map of ORM1-N17A mutant.

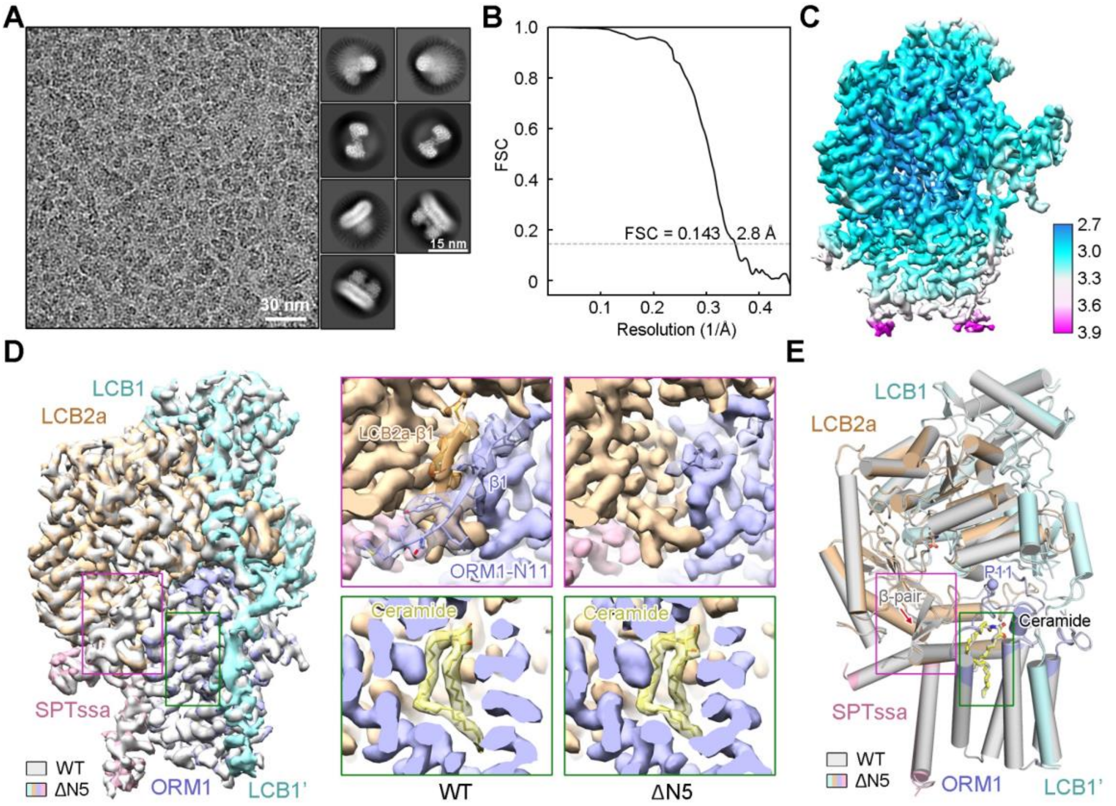

Fig. S13 | Cryo-EM analysis of the SPT-ORM1 (LCB2a-ΔN5) complex. A, Representative cryo-EM micrograph and 2D class averages. **B,** Gold-standard FSC curve for LCB2a-ΔN5 mutant generated using Relion 3.0. **C,** Local resolution map of LCB2a-ΔN5 mutant. **D,** Comparison of the EM maps of WT SPT-ORM1 and LCB2a-ΔN5 mutant. The WT SPT-ORM1 map is colored gray; the LCB2a-ΔN5 mutant map is colored based on the subunits. Insets: Comparison of the EM maps for the N-terminal hybrid β-sheet between ORM1 and LCB2a (upper) and ceramide (lower) in the WT SPT-ORM1 complex and the LCB2a-ΔN5 mutant. The N-terminal hybrid β-sheet disappeared in the EM map of the LCB2a-ΔN5 mutant. The ceramide-like density remains the same in the LCB2a-ΔN5 mutant. **E,** Comparison of the structures of WT SPT-ORM1 and LCB2a-ΔN5 mutant. Pro11, the resolved N-terminus of ORM1 in the LCB2a-ΔN5 mutant, is shown in a light blue sphere.

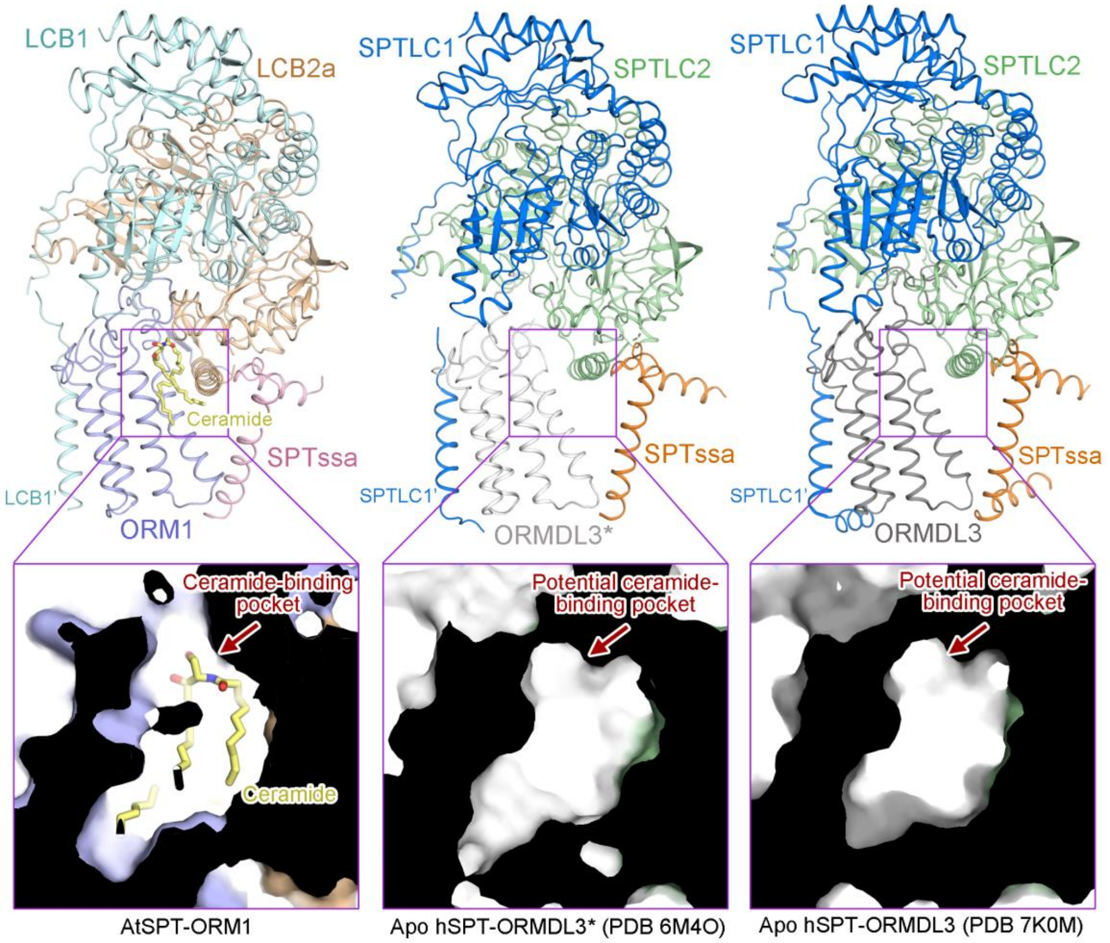

Fig. S14 | Potential ceramide-binding cavity in hSPT-ORMDL3. The hSPT-ORMDL3 complex possesses a similar ceramide-binding pocket between ORMDL3 and SPTLC2 as that in the AtSPT-ORM1 complex.

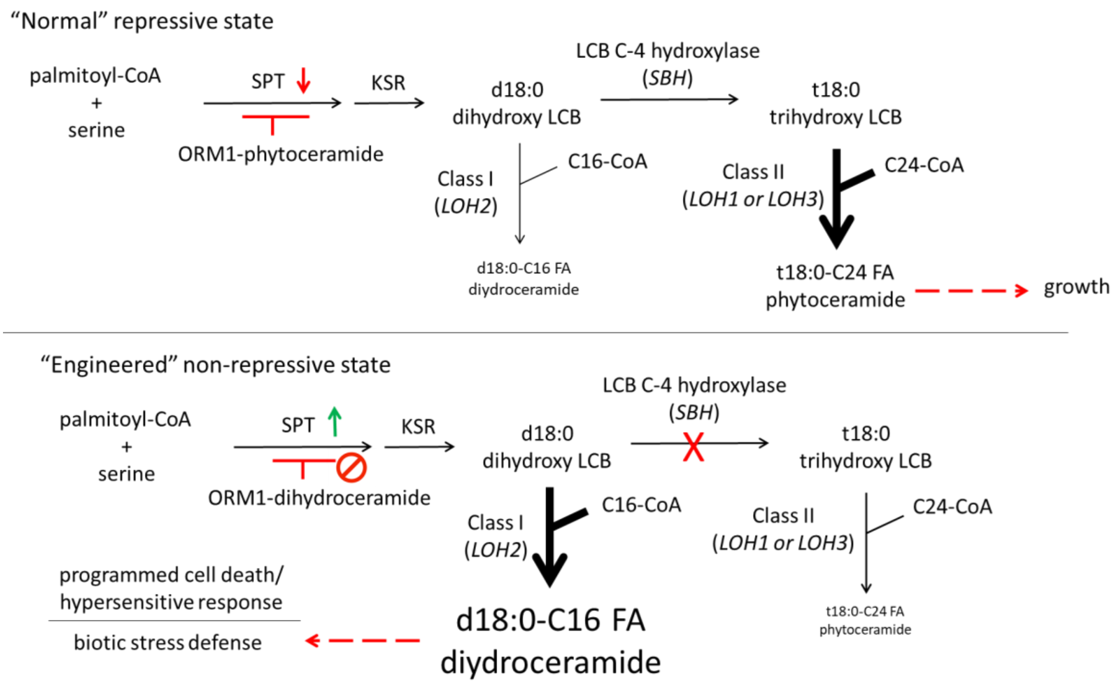

Fig. S15 | Balancing sphingolipid biosynthesis for growth and defense: model showing differential effects of phytoceramides versus dihydroceramides on ORM1-mediated repression of SPT activity. Top panel: Wild-type *Arabidopsis* preferentially produces phytoceramides via *LOH1*- or *LOH3*-encoded Class II ceramide synthases for glycosphingolipids to support growth. As sphingolipid production exceeds the amounts required for growth, excess phytoceramides bind SPT-ORM1 to confer repression of SPT activity. Bottom panel: In *Arabidopsis* engineered for up-regulation of the *LOH2*-encoded Class I ceramide synthase or knockout of *SBH*-encoded LCB C-4 hydroxylase, C16-dihydroceramides accumulate to high levels relative to phytoceramides. Dihydroceramides do not effectively confer ORM1-mediated repression of SPT activity. The result is the unregulated production of LCBs from SPT and dihydroceramides and the triggering of programmed cell death and salicylic acid, hallmarks of the hypersensitive response for plant microbial pathogen defense. SPT, serine palmitoyltransferase; KSR, 3-ketosphinganine reductase; LCB, long-chain base.

Table S1 | Cryo-EM data collection, refinement, and validation statistics.

	SPT-ORM1 dimer/monomer (EMD-33873, EMD-33874; PDB 7YJK, PDB 7YJM)	ORM1-N17A variant monomer (EMD-33875, PDB 7YJN)	LCB2a-ΔN5 variant monomer (EMD-33876, PDB 7YJO)
**Data collection and processing**			
Magnification	130,000	130,000	130,000
Voltage (kV)	300	300	300
Electron exposure ($e^{-}/Å^{2}$)	50	50	50
Defocus range (μm)	-2.0 to -1.0	-2.0 to -1.0	-2.0 to -1.0
Pixel size (Å)	1.08	1.08	1.08
Symmetry imposed	C2/C1	C1	C1
Initial particle images (no.)	801,707	930,704	893,978
Final particle images (no.)	159,298/115,440	58,458	279,059
Map resolution (Å)	3.2 Å	3.4 Å	2.8 Å
FSC threshold	0.143	0.143	0.143
Map resolution range (Å)	3.0-4.2 Å	3.3-4.5 Å	2.7-3.9 Å
**Refinement**			
Initial model used (PDB code)	6M4O	6M4O	6M4O
Model resolution (Å)	3.2 Å	3.4 Å	2.8 Å
FSC threshold	0.143	0.143	0.143
Model resolution range (Å)			
Map sharpening *B* factor ($Å^{2}$)	-116/-93	-128	-81
Model composition			
Nonhydrogen atoms	17,660/8,896	8,550	8,770
Protein residues	2,236/1,120	1,099	1,106
Ligands	4/2	1	2
B factors ($Å^{2}$)			
Protein	43.41/45.84	56.34	38.00
Ligand	34.77/39.33	53.55	22.57
R.m.s. deviations			
Bond lengths (Å)	0.008/0.007	0.006	0.011
Bond angles (°)	0.848/0.771	0.872	0.972
**Validation**			
MolProbity score	1.86/1.80	2.01	1.67
Clashscore	6.69/6.18	9.18	4.97
Poor rotamers (%)	0.21/0.00	0.11	0.00
Ramachandran plot			
Favored (%)	91.78/92.69	91.08	93.88
Allowed (%)	7.77/7.04	8.92	5.94
Disallowed (%)	0.45/0.27	0.00	0.18

Table S2 | IC_{50} values of C6-ceramide/phytoceramide for SPT-ORM1 variants.

Batch	SPT-ORM1 variants	Ceramide analogs	IC_{50} (μM) *
1	WT SPT-ORM1	C6-ceramide	3.525 (2.458 – 5.047)
	WT SPT-ORM1	C6-phytoceramide	0.3202 (0.2507 – 0.4079)
	WT SPT-ORM1	C6-dihydroceramide	N/A
2	WT SPT-ORM1	C6-phytoceramide	0.4355 (0.2813 – 0.6749)
	ORM1-N17A		1.680 (0.6065 – 4.150)
	ORM1-S67R		1.257 (0.9431 – 1.669)
	ORM1-N17A/S67R		N/A
	ORM1-W20R		N/A
	ORM1-W88R		N/A
3	WT SPT-ORM1	C6-phytoceramide	0.5670 (0.4137 – 0.7734)
	LCB2a-ΔN5		0.9905 (0.5477 – 1.754)
	ORM1-ΔN8		1.283 (0.8518 – 1.916)
	ORM1-ΔN14		N/A
	ORM1-ΔN22		N/A

* IC_{50} values were obtained by fitting normalized activity data shown in Figs. 2 and 4 (with a 95% confidence interval). N/A = not available.

Table S3 | Primers used in the study.

Primer	Sequence (5'-3')
AtLCB1-pCAG-1-KpnI-F	CGGGGTACCATGGCTTCCAACCTGGTG
AtLCB1-pCAG-482-XhoI-R	CCGCTCGAGTTAGGACTTCAGCAGCAA
AtLCB2a-pCAG-1-KpnI-F	CGGGGTACCATGATCACCATCCCTTAC
AtLCB2a-pCAG-489-XhoI-R	CCGCTCGAGTTAGTCCAGCTTGATGTC
AtLCB2a-ΔN5-pCAG-6-KpnI-F	CGGGGTACCATGTACCTGACCGCTGTG
AtSPTssa-pCAG-1-NotI-F	ATTTGCGGCCGCATGAATTGGGTGCAGCGC
AtSPTssa-pCAG-56-XhoI-R	CCGCTCGAGTTAGGTCAGGTGGCGTTG
AtORM1-pCAG-1-KpnI-F	CGGGGTACCATGGCTAACCTGTACGTG
AtORM1-pCAG-157-XhoI-R	CCGCTCGAGTCACTTGTCACCGTTGAT
AtORM1-ΔN8-pCAG-9-KpnI-F	CGGGGTACCATGGTGCCTCCTCCTGAC
AtORM1-ΔN14-pCAG-15-KpnI-F	CGGGGTACCATGAACCGCAACACCGAG
AtORM1-ΔN22-pCAG-23-KpnI-F	CGGGGTACCATGTACCCTGGTGTGTGG
AtORM1-N17A-F	CCTGACATGAACCGCGCCACCGAGTGGTTCATG
AtORM1-N17A-R	CATGAACCACTCGGTGGCGCGGTTCATGTCAGG
AtORM1-W20R-F	AACCGCAACACCGAGAGATTCATGTACCCTGGT
AtORM1-W20R-R	ACCAGGGTACATGAATCTCTCGGTGTTGCGGTT
AtORM1-S67R-F	GTGGTGACCTACCACAGATTCCACTGGATGAAG
AtORM1-S67R-R	CTTCATCCAGTGGAATCTGTGGTAGGTCACCAC
AtORM1-W88R-F	TACAACGGTCTGACCAGATGGGAGCAGATGGAC
AtORM1-W88R-R	GTCCATCTGCTCCCATCTGGTCAGACCGTTGTA
